# Improved fragment-based protein structure prediction by redesign of search heuristics

**DOI:** 10.1038/s41598-018-31891-8

**Published:** 2018-09-12

**Authors:** Shaun M. Kandathil, Mario Garza-Fabre, Julia Handl, Simon C. Lovell

**Affiliations:** 1 Division of Evolution and Genomic Sciences, School of Biological Sciences, Faculty of Biology, Medicine and Health, University of Manchester, Manchester, M13 9PL United Kingdom; 2 Decision and Cognitive Sciences Research Centre, University of Manchester, Manchester, M13 9PL United Kingdom; 3 Present Address: Department of Computer Science, University College London, Gower Street, London, WC1E 6BT United Kingdom; 4 Present Address: Center for Research and Advanced Studies of the National Polytechnic Institute (CINVESTAV-IPN), Km. 5.5 Carretera Cd. Victoria-Soto La Marina, Cd. Victoria, Tamaulipas 87130 Mexico

## Abstract

Difficulty in sampling large and complex conformational spaces remains a key limitation in fragment-based *de novo* prediction of protein structure. Our previous work has shown that even for small-to-medium-sized proteins, some current methods inadequately sample alternative structures. We have developed two new conformational sampling techniques, one employing a bilevel optimisation framework and the other employing iterated local search. We combine strategies of forced structural perturbation (where some fragment insertions are accepted regardless of their impact on scores) and greedy local optimisation, allowing greater exploration of the available conformational space. Comparisons against the Rosetta Abinitio method indicate that our protocols more frequently generate native-like predictions for many targets, even following the low-resolution phase, using a given set of fragment libraries. By contrasting results across two different fragment sets, we show that our methods are able to better take advantage of high-quality fragments. These improvements can also translate into more reliable identification of near-native structures in a simple clustering-based model selection procedure. We show that when fragment libraries are sufficiently well-constructed, improved breadth of exploration within runs improves prediction accuracy. Our results also suggest that in benchmarking scenarios, a total exclusion of fragments drawn from homologous templates can make performance differences between methods appear less pronounced.

## Introduction

Determining the three-dimensional structure of proteins from only their amino-acid sequences remains a formidable challenge. Computational methods based on the principle of fragment assembly^1,2^ represent the state-of-the-art in tertiary structure prediction^3^. Fragment assembly methods are based on the principle that local amino-acid sequences favour certain local structural features over others^1,4^. By exploiting these local sequence-structure correlations, a model of the tertiary structure is built by combining fragments for short segments of the target sequence. Promising structures are typically identified using a knowledge-based scoring function.

A key limitation of current sampling methods in fragment assembly is their inability to effectively explore alternative conformations within single runs^5^, a situation made worse by fragment libraries being less enriched for native-like structural features in loop regions^6^. In light of these findings, we develop two new methods for conformational sampling. Our methods combine schemes of structural perturbation and local optimisation, which together allow the search to explore alternative structural states more extensively than standard protocols. We demonstrate that the improved breadth of exploration realised by our methods can lead to more reliable prediction of tertiary structure as compared to the Rosetta AbinitioRelax method^1,7^, a widely used *de novo* structure prediction program.

## Background: Fragment-based heuristic optimisation

All methods for protein structure prediction seek to optimise the value of an energy or scoring function, using some operators that bring about conformational variation in the target protein structure. Fragment insertions form the basis of the conformational variation operator in fragment-based search methods, although some methods make use of moves other than fragment insertion to alter structure. Typically, a pre-set number of fragment insertion attempts is used in a single run of a fragment-based sampling method. The typical number of attempts varies from method to method and default values are usually set based on the results of benchmarking experiments using proteins of known structure.

The fragment insertion operation typically involves replacing the conformational parameters (torsion angles or Cartesian coordinates) of a section of the structure with the values of these parameters present in a fragment in the fragment set. Prior fragment insertions can be partially or completely “overwritten” by subsequent ones^8^. Once a fragment insertion or move has been made by the sampling algorithm, it is either accepted or rejected, based on its impact on the energy or score of the system. Rejection of a move implies that the structure is returned to its state just prior to the rejected move. The process of acceptance or rejection of moves is typically governed by a Metropolis Monte Carlo framework^9,10^. The Metropolis criterion always accepts moves that lower the energy (or score) of a system, but also allows for the occasional acceptance of moves that *increase* the energy of the system. The probability *P* of accepting a move associated with an energy change Δ*E* relative to the previous state of the structure, is given by

1 $$P=\{\begin{array}{cc}1 & \,{\rm{i}}{\rm{f}}\,{\rm{\Delta }}E\le 0\\ \exp (\frac{-{\rm{\Delta }}E}{kT}) & \,{\rm{i}}{\rm{f}}\,{\rm{\Delta }}E > 0\end{array}$$

where *k* is the Boltzmann constant and *T* is the thermodynamic temperature. From Eq. 1, higher temperature settings allow the search to accept moves that lead to larger increases in energy or score, whereas lower settings of temperature favour more energetically conservative moves, which may help the search escape from local minima in the energy landscape. Temperature control is therefore a popular means of controlling search behaviour, for example using methods such as simulated annealing^11^, simulated tempering^12,13^ or replica-exchange Monte Carlo^14–16^.

These three concepts were originally introduced in the context of statistical mechanical simulations (such as molecular dynamics), but have been applied in fragment-based protein structure prediction techniques as well. Rosetta formerly employed a simulated annealing procedure as its overall metaheuristic^1,7^. This has been replaced by a constant-temperature search with occasional transitions to higher temperatures (discussed below). The FRAGFOLD method^2,17,18^ also employed simulated annealing as its metaheuristic, with more recent versions making use of replica-exchange Monte Carlo trajectories^19^. Bowman and Pande^20^ describe the application of simulated tempering to Rosetta and report that this increases the explorative ability of the method, although improved predictive accuracy was not observed. Shmygelska and Levitt^21^ employed modified Rosetta frameworks with both temperature and Hamiltonian replica exchange Monte Carlo (TREM and HREM respectively) and demonstrate that HREM outperforms both standard Rosetta and the TREM framework in terms of locating lower-energy states. QUARK^22^ employs a TREM framework to facilitate conformational exploration, with some of its parameters tuned according to the length of the target amino-acid sequence.

It is also possible to improve exploration using schemes of forced structural perturbation. Under such schemes, some moves (e.g. single fragment insertions) can be transiently accepted while ignoring their effect on energy values. This potentially allows the optimisation procedure to escape local minima, depending on the degree of structural perturbation realised. Examples of fragment-based methods employing schemes of forced perturbation are EdaFold^23,24^ and PLOW^25^. Both of these methods make use of schemes of iterated local search (ILS)^26^. In each iteration of ILS, one or more fragment insertions are performed, ignoring their effects on energy and subsequent moves are performed subject to the usual Metropolis criterion for acceptance, or using greedy optimisation.

We will now describe the fragment-based conformational sampling algorithm in the Rosetta Abinitio approach^1,7^. The methods we develop in this study are built as modifications to Rosetta and we build upon our previous work with this method^5,8^. We use Rosetta as a basis for our implementation owing to its availability, popularity and effectiveness^3,27^, although our approach could easily be adapted to any fragment-based search method.

### Low-resolution conformational sampling in Rosetta

In Rosetta’s AbinitioRelax application, the target sequence is first used to construct a linear conformation with a low-resolution representation of the protein, in which the sidechains are represented by a pseudoatom placed at the centroid of each sidechain. All bond lengths and bond angles are set to ideal values taken from Engh and Huber^28^. In this representation, the only changeable parameters are the three backbone torsion angles *ϕ*, *ψ* and *ω*. The backbone torsion angles for all residues are set to *ϕ* = −150°, *ψ* = 150° and *ω* = 180°. Starting from this conformation, fragment insertions are used to alter the values of the backbone torsion angles, nine or three residues at a time. This is done by copying the values of these angles from those taken from a preselected fragment library.

The search algorithm in Rosetta is a Metropolis Monte Carlo sampling strategy using a mostly constant temperature. In Rosetta, the denominator in the exponent of Eq. 1, *kT*, is specified as a single parameter. The value of this parameter is scaled to typical score values seen in Rosetta runs, so as to allow for a reasonable amount of move acceptance and local energy barrier crossing. By default, the temperature parameter takes a value of 2 *kT* units. The temperature is increased if 150 consecutive fragment insertions have been attempted without any move being accepted and this process can occur more than once. Temperature is increased in steps of 1 *kT* unit. Once a move has been accepted, the temperature parameter is reset to 2 *kT* units, regardless of the higher temperature value reached. Such a scheme is sometimes referred to as quenching. The quenching scheme differs from previous implementations, which employed simulated annealing^1^ and is a means for the search to escape from local minima.

Fragment-based conformational sampling in Rosetta is implemented in four stages, each making use of different scoring functions and, in the case of the fourth stage, different fragments. The scoring functions are denoted *score0* through *score5* and are simply different combinations of weights for the terms in Rosetta’s low-resolution scoring function^8^. Each stage has a specified default budget of fragment insertion trials; these are summarised in Table 1. The number of fragment insertion attempts can be increased using a parameter called increase_cycles, which multiplies the default budgets in each stage. Stages 1 to 3 employ 9-residue fragments, whereas stage 4 employs 3-residue fragments.

**Table 1:** Summary of the four stages of the Rosetta low-resolution protocol, with the default number of fragment insertion (move) attempts.

Stage	Default number of move attempts	Termination criterion
Stage 1	2000	Every residue altered once, or maximum number of move attempts exceeded
Stage 2	2000	Maximum number of move attempts exceeded
Stage 3	10 substages of 4000	Convergence detected, or maximum number of move attempts exceeded
Stage 4	4000 (regular moves) + 8000 (Gunn-type moves)	Maximum number of move attempts exceeded

Each stage can terminate once these numbers of attempts have been made, or once other criteria have been met. The default number of move attempts can be altered using a multiplier, increase_cycles. Details of the different move types and termination criteria are given in the main text. In Rosetta, the number of move attempts is equal to the budget of scoring functions.

In stage 1, the sampling process starts from the linear conformation and attempts fragment insertions until every residue has had its torsion angles changed at least once, or after a default of 2000 insertion attempts. Stage 1 can be considered a conformational “randomisation” step, starting from a constant, extended conformation^5^. In stage 2, a default of 2000 insertion attempts are used. Stage 3 is divided into 10 sub-stages, each using a default of 4000 insertion attempts. One of two scoring functions are used in alternation in each sub-stage. Stage 3 also employs a convergence check which can cause the current sub-stage to terminate before all insertion attempts have been used. The convergence check measures the structural similarity of the current structure state to a reference one that is regularly updated. If the search is found to not cause enough variation in the structure after 100 accepted fragment insertions, the current substage is terminated.

In stage 4, 3-residue fragments are used, together with two fragment insertion operators. The first 4000 insertion attempts in stage 4 employ the standard fragment insertion operation as discussed above. However, the last 8000 attempts employ a version of the insertion operator that takes into account the structural variation introduced by a proposed fragment insertion, by calculating a penalty called the Gunn cost^29^. The Gunn cost essentially estimates the lever-arm effect of a proposed fragment insertion and the Gunn cost is minimised when making a fragment insertion in the latter parts of stage 4. Stage 4 does not employ a convergence checking mechanism. Following stage 4, the low-resolution protocol is complete and all-atom refinement can optionally be applied to produce a candidate structure with all sidechain atoms.

Although the search method in Rosetta has components aimed at realising effective conformational sampling, we have previously shown that individual runs of Rosetta can easily become trapped in local minima in the energy landscape^5^, behaviour that can hamper effective structure prediction for many targets. In this study, we build upon the insights from our previous work and propose two new sampling protocols aimed at enhanced exploration of alternative conformations for a target.

## Methods

### Proposed sampling protocols

Our two protocols are variations of a single framework (Algorithm 1). Starting from an initial structure *P*_0_ originating from stage 1 of the low-resolution protocol, exactly as in standard Rosetta (line 1 in Algorithm 1), both methods employ alternating steps of structural perturbation and local search (*Perturbation* and *LocalSearch*, respectively). The exact forms of these operators are specific to each method. In general, the *Perturbation* steps are designed to allow the optimisation process to escape local energy minima by performing relatively large moves in conformational space and accepting the perturbed structure regardless of its energy. The *LocalSearch* steps locate a local minimum near the perturbed structure, following the local energy gradient, using a scheme of greedy optimisation. The *Perturbation* steps make more disruptive changes to the protein structure, following which the *LocalSearch* steps can make small alterations to this perturbed structure, for example, to alleviate bad atomic clashes. The strategy of alternating perturbation and local search is sometimes referred to as basin-hopping, reflecting the fact that the perturbation steps are designed to allow the search to escape from local minima in the energy landscape, following which the local search steps allow gradual descent into alternative basins. In Algorithm 1, structural states corresponding to local minima are denoted *LMin* and *LMin*′. Given the multimodal nature of the scoring functions, local minima encountered during the search may be structurally different. Therefore, all local minima encountered during the search are considered for addition to an archive of promising solutions (lines 4 and 8 in Algorithm 1) and the archive of solutions is output at the end of the entire procedure. Currently, the archive simply stores the 10 lowest-scoring *LMin*s encountered at any time (further details in Supplementary information). Through the comparison of successive local minima (*AcceptanceCriterion* in line 9), the algorithms are able to explore many minima in the energy landscape and make a more informed decision of a conformational neighbourhood in which to settle. *AcceptanceCriterion* compares energy or score values of consecutive *LMin*s using the usual Metropolis criterion, where the temperature parameter is set by a scheme of simulated annealing (details in Supplementary information). Thus, more disruptive conformational changes are allowed near the start of the optimisation process and more conservative changes are favoured towards the end. The entire procedure is terminated after a certain number of calls to the scoring functions have occurred (line 10 in Algorithm 1); this is set in order to maintain comparability with runs of standard Rosetta. Additional details of the implementation of the various components of our protocols are given in the Supplementary Information.

**Algorithm 1:**
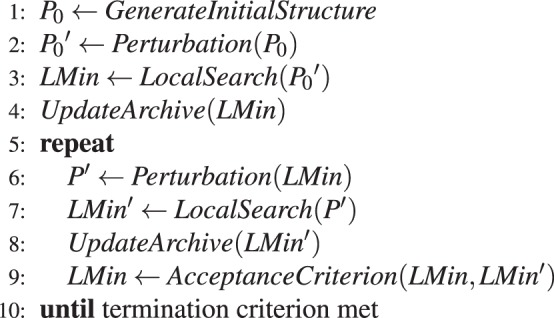
General scheme of proposed sampling protocols.

#### Bilevel protocol

Our first sampling strategy uses bilevel optimisation^30,31^, which partitions the variables to be optimised into two sets, with an optimiser operating on each such subset of decision variables. First, an “upper-level” optimiser sets the values of its subset of decision variables. The values of these decision variables are then held fixed while the “lower-level” optimiser modifies its set of decision variables. In this way, the setting of the upper-level decision variables are used as a constraint for the “lower-level” optimisation problem. Once the lower-level optimiser has reached a minimum, the next iteration can begin with the upper-level optimiser making another change to its decision variables.

In our implementation the *Perturbation* and *LocalSearch* steps operate on distinct subsets of residues, altering backbone torsion angle triplets using fragment insertion operations as implemented in Rosetta. Previously we demonstrated that loop regions are difficult to sample using typical approaches^5^. As fragment libraries are less enriched for native-like structural parameters in these regions^6^, we hypothesised that the use of a bilevel framework, in which the torsion states of loop regions are treated as upper-level decision variables, may increase fold-level exploration. Therefore, in our bilevel framework, the *Perturbation* steps are allowed to alter the values of torsion angles in residues predicted to be in loops, while the *LocalSearch* steps operate on non-loop residues. The *LocalSearch* operator thus corresponds to the lower-level optimiser, whereas the upper-level optimiser comprises all steps in lines 2 to 10 in Algorithm 1 except the *LocalSearch* steps. Secondary structure (SS) predictions from PSIPRED^32^ inform this assignment and rules are defined for the application of a *Perturbation* step, depending on the local SS assignment in a fragment insertion window (Supplementary Table S1), whereas the *LocalSearch* steps operate only on non-loop residues. These rules mean that the fragment insertions performed in the *Perturbation* and *LocalSearch* steps do not always affect nine residues at a time. We expect that the combination of moves operating separately on loop and non-loop residues should allow for systematic exploration of different arrangements of SS elements and so encourage conformational exploration.

#### ILS protocol

Our second method employs iterated local search (ILS)^26^. In ILS, a local search operator (or procedure) is also applied in alternation with perturbation steps, but in ILS both the *LocalSearch* and *Perturbation* steps affect the entire set of decision variables. In other words, two different optimisation strategies can be employed without one constraining the other in any way and so the perturbation and local search steps are allowed to affect all parts of the protein structure. ILS is, therefore, a generalisation of the bilevel optimisation approach. It represents a more basic implementation of basin-hopping, which has been described in a fragment assembly context^24,25^, although this has not previously been combined with a simulated annealing scheme for comparing successive local minima.

#### Implementation

Our protocols are implemented by replacing stages 2 and 3 of the low-resolution protocol in Rosetta’s AbinitioRelax application (version 3.4). Low-resolution stages 1 and 4^7^ remain unchanged. Stage 1 was not altered since this acts merely as a structural randomisation step^5^. We disable Rosetta’s convergence checking mechanism in stage 3 and we apply Rosetta’s standard stage 4 separately to each solution stored in the archive of low-energy solutions, following stage 3. This allows the search to arrive at more compact structures for the archived solutions, through a more exploitative search. This is because stage 4 uses more conservative insertion operators that take the structural change introduced by the particular insertion (Gunn cost) into account^7,29^, in addition to standard 3-mer fragment insertions. In preliminary tests, we found that applying our new search operators in stage 4 often led to broken secondary structure elements and structures that lacked compactness (data not shown). This setup enables broad conformational exploration (stages 1 through 3), with methods that realise local energy minimisation (stage 4). Trajectories of the bilevel and ILS protocols were run as single longer runs, with the increase_cycles parameter set to 100. Since stage 4 is applied to each archived structure, we reduce the length of stage 4 by a factor of 10, so as to keep the number of scoring function evaluations used per decoy constant between Rosetta and our protocols. The default budgets of scoring function evaluations can be found in the paper by Rohl *et al*.^7^.

### Targets and fragment sets

Predictions were made for 59 small-to-medium-sized protein domains, used in our previous work^5^. We used the same fragments as used in that study and also used older fragment libraries, to study the effect of fragment library quality on predictive accuracy. The newer fragments were generated using the fragment picker and the *vall* structure database supplied with Rosetta version 3.5 (weekly release 2014.16.56682). The NCBI non-redundant sequence database used during fragment picking was downloaded on April 29, 2014. We used PSIPRED version 3.3 as the sole SS prediction method informing the fragment generation process. Identical fragment libraries were supplied to all sampling protocols being compared and homologous template structures were excluded using the standard method in the Rosetta fragment picking pipeline, by specifying the -nohoms argument to the make_fragments.pl script. All three protocols made use of the 25 highest-scoring 9-mer fragments and the top 200 3-mer fragments. In the case of the bilevel protocol, the PSIPRED predictions generated during fragment picking were used to define loop and SS element boundaries. The older fragment libraries were available to us from a previous study^8^ and were generated no later than November 2007. PSIPRED version 2.5 was used during the generation of these fragments. When either old or new fragments were used with the bilevel protocol, the PSIPRED prediction derived during the generation of the respective fragment set was used. A number of current protocols use fragments of various lengths^22,33^. Here, we limit our fragment sets to lengths of 3 and 9, corresponding to the “traditional” settings and what most users of Rosetta will use e.g. if fragments are obtained from the Robetta server^34^. This also affords some comparability with results in other published work^5,8,23,24^.

### Local and global measures of sampling

We will compare the performance of our protocols against Rosetta’s AbinitioRelax application (version 3.4). Since sets of shorter Rosetta runs provide superior performance as compared to single, longer runs^5^, we will compare our protocols against sets of short Rosetta runs for all experiments in this work.

We evaluated trajectories of Rosetta and our protocols using our local and global analysis techniques, described previously^5^. For *local* measures of sampling, we examined the number of accepted changes per residue, as well as the fraction of unique backbone torsion angle triplets sampled per residue. The former is simply a count of the number of times the backbone torsion angles for each residue were changed during the search, while the latter considers what fraction of the set of unique torsion angle triplets available in the fragment set at each residue position were sampled at least once. These evaluations, in isolation, are not a measure of conformational exploration (they do not consider whether conformations are protein-like), however they are intended to assess whether different parts of the chain are adequately sampled. For the bilevel and ILS protocols, we took into account all moves that were accepted by the search, including perturbation and hill-climbing steps that were subsequently discarded by the search following the comparison of successive *LMin* structures (see Section “Proposed sampling protocols”). Our rationale for counting these moves is that they correspond to useful exploration, even if they were only transiently accepted, since the information they provide is used to make decisions during the search. This is in contrast to Rosetta, for example, in which decisions about move acceptance are made after each fragment insertion. For the local analyses, we analysed 20 independent trajectories of Rosetta and our methods. Rosetta was run as 20 sets of 10 short runs and each set of 10 runs used the same budget of scoring function evaluations as one run of our protocols.

Our *global* measures of sampling assess the extent to which individual runs visit distinct conformational states for a given target. We employed our multidimensional scaling (MDS) method for visualising the movement of trajectories through conformational space, as well as our measure based on Shannon entropy for quantifying exploration, described previously^5^. Both of these global measures of sampling characterise the breadth of sampling achieved by single trajectories of a structure prediction method. This is achieved by comparing states sampled on individual trajectories to a previously obtained sample of decoys, the latter representing the available conformational space for a target protein. The MDS procedure visually represents this data by means of dimensionality reduction and can be used to visualise the movement of prediction runs through conformational space, since more similar structures will be plotted closer together. MDS projections were obtained using the cmdscale function in R version 3.3.0, which implements classical MDS for non-Euclidean input dissimilarity matrices. In our case, these dissimilarities are the Hamming distances between the binary contact maps of the structures used in the analysis^5^. Once a dissimilarity matrix has been composed, dimensionality reduction in classical MDS is achieved by (a) eigendecomposition of the input dissimilarity matrix after squaring and double centering and (b) construction of new coordinates by multiplying the square root of the *k* largest positive eigenvalues with the corresponding eigenvectors. Step (b) gives the *k* principal coordinates that individually capture the most variation in the data. We use *k* = 2 in all our MDS analyses. A detailed description of classical MDS can be found in Chapter 12 of Borg and Groenen (2005)^35^.

Our entropy measure allows us to quantify and compare explorative behaviour between methods and uses the same data as used for the MDS representation (without any form of dimensionality reduction). Higher values of the entropy measure indicate that a method frequently visits a good fraction of the available conformational space for a given target, whereas low values indicate trapping in local minima. The entropy measure is calculated by clustering all the trajectory data along with the set of decoys for a given target protein and modelling the movement of individual trajectories through the clusters as a Markov state model. A modified version of the Shannon entropy^36,37^ is then used to quantify exploration.

For both global analyses, 8 independent trajectories of each sampling method were analysed, together with a sample of 1000 Rosetta low-resolution decoys. The latter defines a sample of the compact conformational states for each target and is used to evaluate the extent to which each trajectory explores the available space for that target, as defined by the fragment library. To ensure that the native basin was represented in the structure set, the native structure was also included.

For Rosetta, we sampled the folding structure after every 100th accepted move during each trajectory. We analysed 8 sets of 10 short runs, excluding structures from stage 4, since the bilevel or ILS strategy is only active in low-resolution stages 2 and 3. This lets us compare the performance of these sampling strategies against the equivalent parts of Rosetta and decreases the complexity of the resulting MDS plots. For our protocols, we recorded every 100th *LMin* generated during each trajectory. We sampled structures from *LMin*s only, since this describes the extent to which different compact states are sampled. Recording the state of the protein, say, every 100th accepted move (as done for Rosetta) can lead to trajectories in which large portions of the sampled structures correspond to less-compact, unfolded states. This is a result of our scheme of forced perturbation, which can introduce very disruptive moves. Including perturbed states can lead to overestimation of the extent of useful exploration realised, since many of these structures are infeasible.

To calculate values of our entropy measure, trajectory points for each protein and protocol were clustered with a set of Rosetta decoys, as done for the MDS procedure above. The PAM method^38^ was used to cluster these data into 20 clusters. Entropy was calculated from the clustering solution for each of the 8 trajectories of each method and differences between methods were assessed using the Mack-Skillings statistical test^39^. The “conservative” version of the post-hoc procedure was used^40^, with an experiment-wise error rate of *α* = 0.05. The critical value of the test statistic *S* used in the post-hoc procedure was calculated using 50,000 iterations of Monte Carlo simulation.

### Assessment of predictive accuracy

For assessing predictive accuracy, we ran 1000 runs of Rosetta with the increase_cycles parameter set to 10, corresponding to the typically-employed strategy of running many short runs. This number of runs was chosen such that RMSD distributions generated do not vary significantly between sets of runs (data not shown). For the bilevel and ILS protocols, we ran 100 independent runs with the increase_cycles parameter set to 100. We used our archiving strategy to store 10 of the lowest-energy solutions seen during each run. Thus, a set of 100 runs with any of the protocols returns 1000 decoy structures in total. To compare RMSD distributions, we used kernel density plots with a Gaussian kernel and a constant bandwidth of 0.7 Å, drawn using the beanplot package^41^ for R 3.3.0.

### All-atom refinement and model selection by clustering

We used the standard Rosetta *FastRelax* procedure^42^ to generate compact all-atom structures. We ran 50 rounds of *FastRelax* with default parameters for each low-resolution decoy generated by Rosetta and our protocols, giving a total of 50,000 all-atom decoys per target protein and protocol. These decoys were then clustered using Calibur^43^ to identify promising solutions, based on pairwise Cα RMSD between decoys. We used Calibur instead of Rosetta’s own clustering application, since Calibur provides greatly improved time complexity on large decoy sets, without degradation in result quality in benchmarks^43^. This is achieved by the use of prefiltering strategies which rapidly exclude decoys from consideration, avoiding the need to compare every pair of decoys. Default parameters were used for Calibur, which includes automatic determination of RMSD clustering thresholds. Calibur reports the centres and the members of the three largest clusters found. We make assessments on the top-1 and top-3 decoys listed by Calibur. These are the cluster centre of the first and the centres of all three reported clusters, respectively.

## Results

### Improved explorative ability

**Figure 1:**
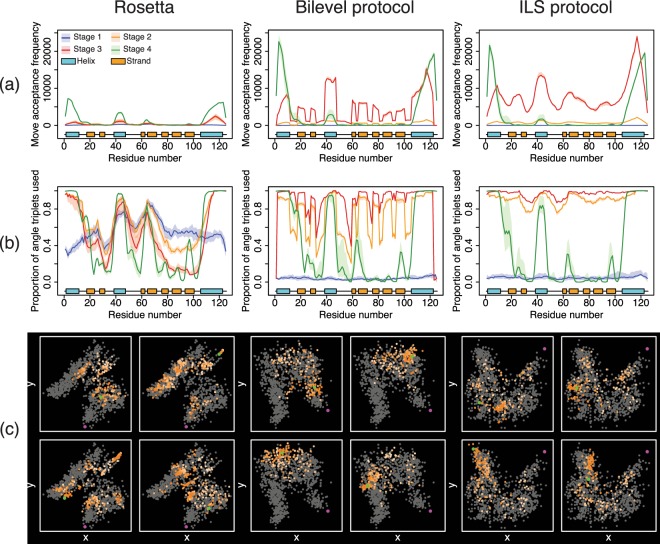
Local and global measures of sampling, for one target (PDB ID 1acf, 125 residues, *α* + *β*). Entropy data is given in Fig. 2. Row (**a**): number of accepted changes per residue, for each stage of the low-resolution protocol (stage 1 in blue, stage 2 in orange, stage 3 in red and stage 4 in green). Values are represented as medians (*n* = 20) and the shaded regions represent the interquartile range. Predicted secondary structure is beneath each plot: *α*-helices are in blue and *β*-strands are in orange. Row (**b**): Unique torsion angle triplets available per residue sampled at least once in the search. Data are represented as median and interquartile range using the same colour scheme as row (**a**). Row (**c**): Multidimensional scaling (MDS) plots for four runs of each protocol. Grey points represent a constant set of 1000 Rosetta decoys. Points sampled from trajectories are coloured orange with deeper colours towards the end of a run. A single green point represents the endpoint of the trajectory and the native structure is purple.

Our protocols are designed to improve conformational sampling by improving the extent to which certain protein regions (in particular, loops) vary structurally during each run. Row (a) in Fig. 1 compares the frequency of accepted moves per residue in the stages of the low-resolution protocol in Rosetta and our protocols, for the target 1acf. It can be seen that in stages 2 and 3 (orange and red lines, respectively), where our sampling methods are active, the frequency with which fragment insertions are accepted is much higher. These trends correlate with increased exploration of the available backbone torsion triplets as well (Row b) and are typical for all proteins in our dataset. These results suggest that single, long runs of our protocols are capable of comparable or more extensive conformational exploration relative to sets of short Rosetta runs.

For the bilevel protocol the values of both local measures vary considerably with secondary structure. This reflects the nature of the optimisation strategy used: the *Perturbation* steps employ a single fragment insertion that is only allowed to alter loops and the subsequent *LocalSearch* steps attempt a series of moves that are allowed to alter SS elements only. Thus, the loops show a relatively reduced number of accepted moves, relative to the SS elements. Terminal loop regions display zero values of our local measures in stages 2 and 3 as they are not altered by either the *Perturbation* or the *LocalSearch*.

In contrast to the bilevel protocol, the ILS protocol displays a higher number of accepted moves in the loop regions and termini, particularly in stage 3. This correlates with an improved fraction of torsion angle triplets explored per residue as well, because ILS has fewer restrictions on where a move can be made, in both the perturbation and *LocalSearch* steps. For example, the ILS protocol can accept fragment insertions in loops as part of the *LocalSearch* steps, if these moves immediately lead to better score values, whereas the bilevel protocol cannot. This is expected: if the *LocalSearch* steps in the bilevel protocol affected loop regions the constraints set up by the *Perturbation* step would be violated.

**Figure 2:**
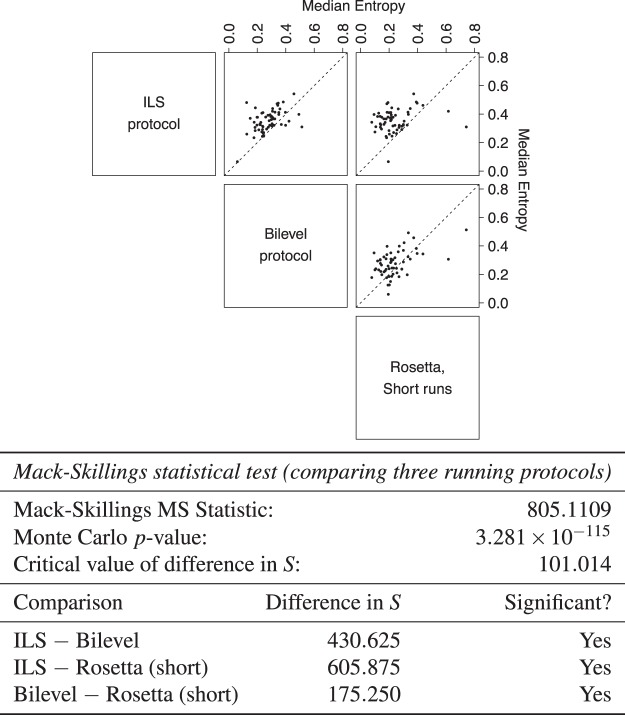
The ILS protocol shows significantly higher entropy than all other protocols. Top panel: Pairwise scatterplots comparing median entropy values for 8 trajectories of Rosetta and the bilevel and ILS protocols, for all target proteins. Dashed lines of unit slope are drawn. Bottom panel: Results of statistical testing of entropy data. Values for all 8 independent runs of each protocol were compared. Mack-Skillings (MS) test statistics, *p*-value and post-hoc pairwise comparisons are shown. The entropy associated with any two protocols are significantly different if the magnitude of the difference in the measure *S* for these protocols is greater than the critical value shown. The sign of the difference indicates which protocol typically shows higher entropy.

The global measures of sampling indicate that our protocols better explore the available conformational space within individual runs (Row c in Figs 1 and 2). From Row c in Fig. 1, considering the trajectories of our protocols, the tendency of rapid convergence to a single localised area of conformational space that we observe within individual Rosetta runs is absent. Instead, the local minima encountered during the search are spread among distinct areas of conformational space. This is indicated by the points sampled in single trajectories (orange dots) covering a good proportion of the available conformational space for this target (grey points). This effect is most noticeable on more difficult targets (such as 1acf), for which individual Rosetta runs quickly descend into local minima in the energy landscape, indicated by the localised clustering of points corresponding to individual trajectories in the data for Rosetta. Data for Rosetta are obtained by concatenating structures from 10 short trajectories. Since the data plotted in orange points are taken from single trajectories (or sets of trajectories in the case of Rosetta), the final structure need not be located close to the native. Instead, the plots show only the overall explorative behaviour of the trajectories.

On targets such as 1acf, single runs of our protocols appear to realise a comparable degree of exploration as sets of short Rosetta runs. This result is encouraging and indicates that our protocols have lower susceptibility to local minima in the energy landscape, within single longer runs. This behaviour allows us to exploit the diverse nature of the local minima sampled within trajectories and guide sampling towards native-like states. In contrast, sets of short Rosetta runs share no information between them and thus cannot influence each other. In our protocols, we use our archive of promising solutions (Section “Proposed sampling protocols”), to decide whether newly encountered local minima should be retained, by comparing these to a set of such minima that were previously encountered. In this manner, we make use of more information to guide the search, as compared to a set of Rosetta runs.

The MDS analyses provide an approximate and visual method to analyse search behaviour; it is desirable to have a quantitative measure of explorative ability without relying on dimensionality reduction and this is what our entropy measure provides. The top panel of Fig. 2 compares median values of our entropy measure for trajectories of Rosetta and our protocols, for all the targets in our data set. A tabular form of this data is given in Supplementary Table S2. The ILS protocol typically achieves a significantly higher entropy than the bilevel protocol and Rosetta (Fig. 2, bottom panel), in agreement with our local measures, highlighting its improved exploration ability. Although entropy data is best compared between protocols for which trajectory data have been sampled in a similar manner, taken together, the results from our local and global measures of sampling do point to an increased degree of conformational exploration in runs of our protocols.

### Native-like structures are more frequently accessed

**Figure 3:**
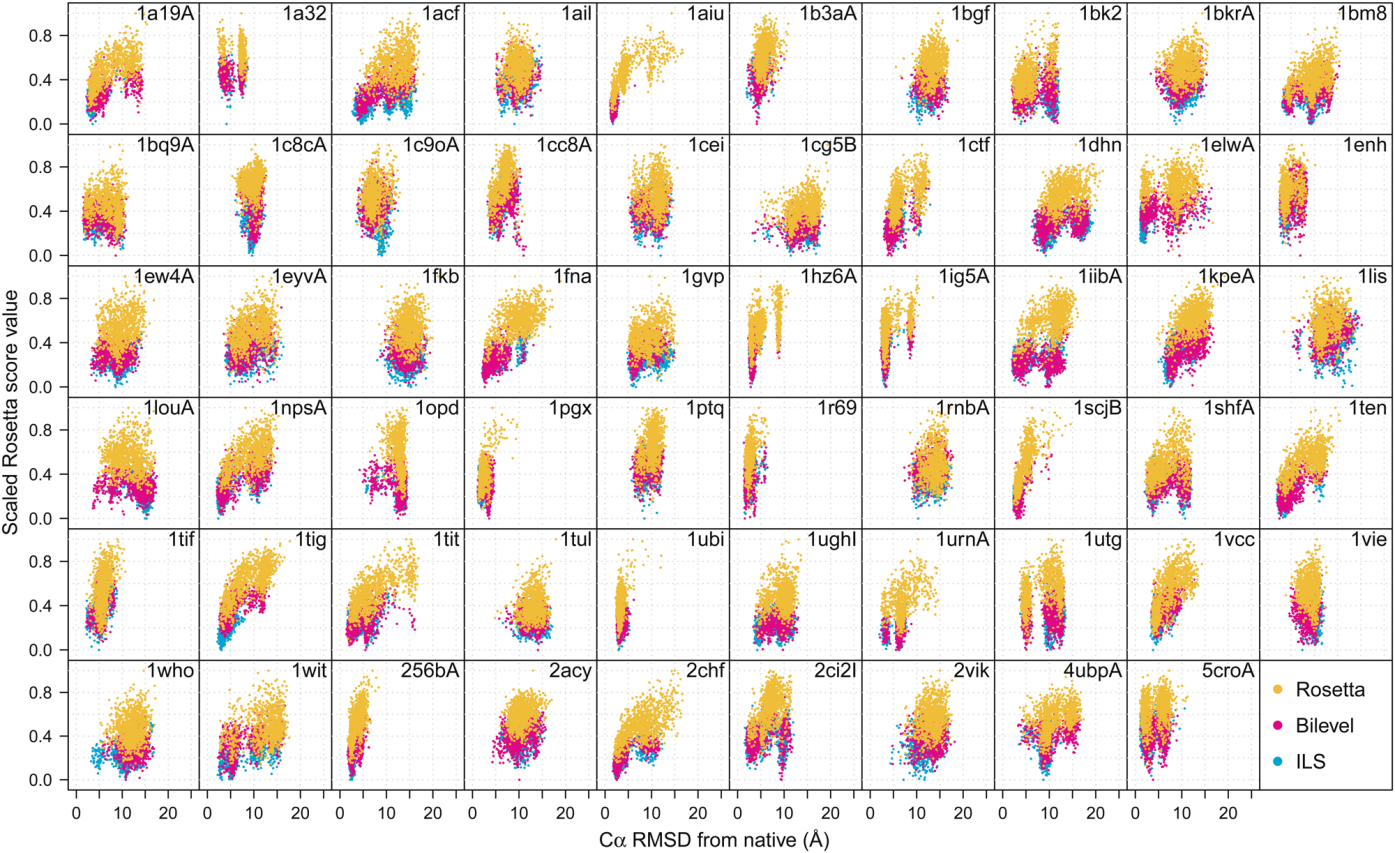
Scaled Rosetta score values following the low-resolution phase (*y*-axis) versus Cα RMSD from the native structure for all 59 target proteins in our dataset. Score data are scaled to the range [0,1]. Points correspond to 1000 low-resolution decoys generated by standard Rosetta, the bilevel protocol and the ILS protocol, using newer fragment libraries. The bilevel and ILS protocols show improved predictive ability on many targets, indicated by larger numbers of points in the low-score, low-RMSD regions of the plot. RMSD distributions are shown in red in Supplementary Fig. S2. An extended version of this data is available on Zenodo (see Methods and Data availability), which also includes data obtained with the older fragment sets.

It is almost trivially easy to modify search protocols to achieve a higher degree of exploration, however this is not useful if no protein-like conformations are generated and retained. The ultimate aim in developing novel sampling protocols is to try and improve the accuracy of the resulting predictions. Figure 3 compares the distributions of low-resolution Rosetta scores and Cα RMSD values obtained by runs of our protocols and short runs of standard Rosetta, over 1000 low-resolution decoys. On many targets, one or both of our protocols display a marked improvement in the frequency with which near-native structures are identified as compared to Rosetta, in the low-resolution phase. Taken together with our local and global measures, this result suggests that our protocols are able to reach near-native conformations more reliably as a result of improved conformational exploration. Importantly, we are able to realise these distributions using a comparable budget of scoring function evaluations as a typical Rosetta protocol. This suggests that accurate *de novo* prediction may be possible with a relatively small number of replicate runs, provided that each run is capable of effective exploration of the search space.

The performance of our protocols on some targets suggests that (at least for these targets) the scoring functions provide sufficient guidance to help reach the native basin and that the fragment sets for these targets support native-like structures. The inferior performance of Rosetta on these targets suggests that the availability of native-like fragments in the fragment set is such that a more advanced sampling technique is necessary in order to exploit these. We revisit this key point in more detail in section “Improved utilisation of high-quality fragment sets”.

The use of SS information to inform the moves in our bilevel protocol does not appear to improve explorative ability to the same degree as the ILS protocol. Predictive performance is typically comparable for our protocols, with the ILS protocol usually performing slightly better when considering distributions of RMSD values. This suggests that the source of the improvements in predictive accuracy of our protocols appears to be the combination of perturbation and greedy local minimisation that both of our methods employ, rather than the particular type of perturbation operation employed. A range of factors could be responsible for the differences in performance between the bilevel and ILS protocols, including inaccurate secondary structure predictions (an example is given in the Supplementary material), or the fact that fragments are less likely to be native-like in loop regions^6^.

For targets with no improvement in RMSD relative to Rosetta, our protocols are typically no worse, although targets 1louA and 1utg are exceptions. Since our protocols optimise score values, certain structural states would be accessed frequently if they were frequently represented in the fragment libraries and if they were favoured by the scoring functions. In other words, if the fragment libraries strongly favour a set of low-scoring (but incorrect) topologies for the target protein, these are the structures that will most frequently be accessed by our protocols. This also implies that our protocols are more susceptible to inaccuracies in the scoring functions. These limitations could be overcome through the use of alternative decoy archiving strategies, which can encourage greater structural diversity in the solution set.

Although the standard homologue detection and exclusion procedure was employed during fragment generation (See Methods), we found that homologous template structures provided fragments for many targets, including some targets for which predictive accuracy was poor with all three protocols (e.g. targets 1dhn and 1cg5B). This is likely due the fixed set of PSI-BLAST E-value cutoffs used to identify homologues during the fragment picking process, regardless of target sequence^44^ and E-values are known to depend on database size and query length. Thus, a cutoff that identifies homologous sequences for one target sequence may miss homologues for another sequence. Despite the presence of some fragments from homologous templates in the fragment libraries, Rosetta has not improved the sampling of near-native structures in many cases, even though the same fragment sets were supplied to all three methods. This suggests that improved exploration of the search space and improved optimisation of low-resolution scores can improve predictive accuracy. There are also some targets in our dataset for which our protocols achieve markedly better results than Rosetta, even though we found no evidence of fragments from homologous templates (e.g. targets 1a19A, 1acf, 2ci2I and 2chf), suggesting that fragments from homologues are neither necessary nor sufficient for successful prediction. Near-native structures are not always sampled even when homologous fragments are present (e.g. targets 1dhn and 1cg5B), which suggests that a certain fraction of the conformational space may need to correspond to the native basin for it to be accessed reliably. Poor accuracy for some targets even in the presence of fragments from homologues could also indicate that the scoring functions are inaccurate^20,45^, although this is problematic to assert without first showing that given search methods can actually *generate* native-like conformations using a given fragment set.

### Results following refinement and model selection

For our methods to be truly useful in protein structure modelling, the low-resolution decoys returned by our methods should serve as good starting points for all-atom refinement. The predictive accuracy distributions seen after the low-resolution phase are retained following refinement (Supplementary Fig. S1), suggesting that our low-resolution decoys are reasonable starting points for refinement procedures.

**Figure 4:**
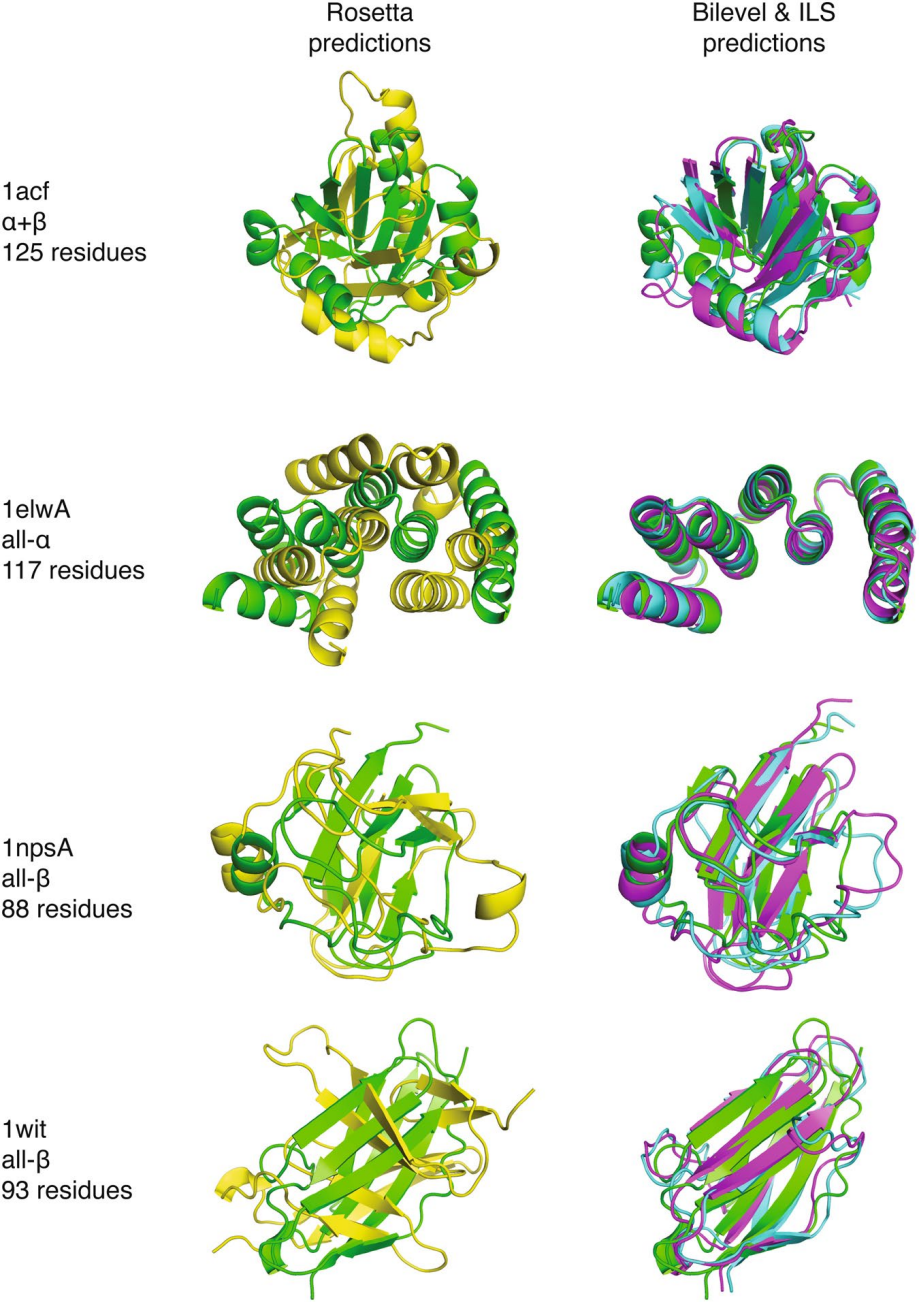
Examples of top models identified by clustering having improved accuracy. The top cluster centre identified from decoy sets generated by Rosetta (yellow), the bilevel (magenta) and ILS protocol (blue) are shown superimposed on the native structure (green) for 4 targets. For the targets shown, our protocols sample an improved number of decoys near the native and this translates into the clustering procedure identifying more native-like structures as the most promising. Complete distributions of RMSD values are given in Supplementary Fig. S1 and RMSD values of top cluster centres are given in Supplementary Table S3.

For targets with improvements in low-resolution RMSD distributions, we often find that the top cluster centre selected from the bilevel and/or ILS decoy sets has greatly improved predictive accuracy compared to that obtained by Rosetta (Fig. 4). Improving the frequency with which near-native solutions are accessed at the low-resolution level has the potential to improve results following all-atom refinement and clustering. In spite of these successes, however, when the whole set of targets is considered, we find that the top cluster centres from the decoy sets generated by our protocols are broadly comparable in quality to that chosen from the corresponding Rosetta decoy sets (Mack-Skillings test, *p* = 0.157). Supplementary Table S3 shows the Cα RMSD values of the best cluster centre chosen by Calibur, for all targets and protocols. Differences between Rosetta and our protocols become significant when the top three cluster centres are considered (*p* = 0.00027).

### Improved utilisation of high-quality fragment sets

**Figure 5:**
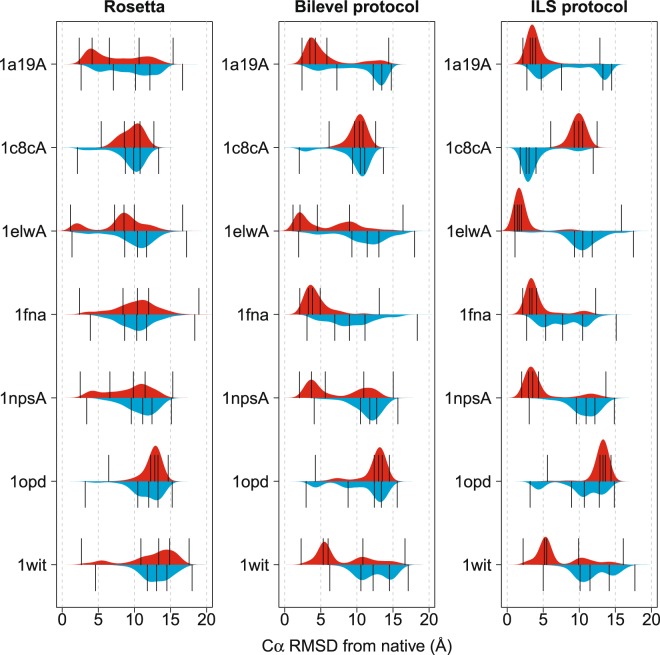
Kernel density plots of Cα RMSD from the native structure, comparing decoy sets generated using the older and newer fragment libraries (blue and red distributions, respectively), for Rosetta, the bilevel protocol and the ILS protocol (left, centre and right panels, respectively). Each distribution comprises data from 1000 low-resolution decoys sampled using identical scoring function counts. Vertical lines represent the minimum, first quartile, median, third quartile and maximum of each distribution. Our protocols more reliably locate native-like solutions when high-quality fragment sets are supplied (indicated by a shift in RMSD distibutions towards lower values), whereas Rosetta’s distributions do not improve to the same degree. With the older fragments for the target 1c8cA, the ILS protocol performs significantly better than the bilevel protocol; this is due to inaccurate predicted SS (see Supplementary information).

Fragment library quality affects predictive accuracy. To observe the effects of using different fragment sets, we ran a second set of predictions, using older fragment libraries from a previous study^8^. Figure 5 compares the predictive accuracy obtained by Rosetta and our protocols for seven targets when using newer and older fragment sets, represented by red and blue distributions, respectively. These targets showed the largest differences in prediction accuracy when using different fragment sets. Full data is in Supplementary Fig. S2. Comparing Rosetta’s performance on the newer and older fragment sets might suggest that these fragment sets are of broadly comparable quality in terms of the predictive accuracies typically seen. However, when our protocols are used, pronounced differences can sometimes be seen when the two fragment sets are used. Since these experiments used identical scoring functions and numbers of scoring function evaluations, the only differences between the protocols (for a given fragment set) are the conformational sampling methods they employ. Thus, we conclude that compared to Rosetta, our protocols more reliably access native-like conformations, when the fragment sets are enriched for such states. Predictive accuracy distributions are used to assess fragment library quality^6,44,46^ and there is clearly an interaction between fragment sets and search protocols. Improved fragment quality may be difficult to exploit without effective conformational sampling protocols.

## Conclusions

A key advantage of fragment assembly for protein structure prediction is the great reduction in the size of the conformational space that needs to be searched during the prediction process. This is achieved through the use of fragment sets, which serves the additional purpose of altering the search problem from a search in the continuous space of Cartesian coordinates (or torsion angles) into a combinatorial one, where the aim is to find the set of correct *combinations* of fragments that give native-like conformations. However, possible solutions are still too many to enumerate even for small proteins, and the size of the search space increases exponentially with target size^8^. There is a need for algorithms that can effectively search the vast number of possible conformations and reliably locate native-like solutions. Here, we introduced two new sampling methods to try to achieve this aim. The design choices were informed by our previous analyses of the deficiencies of current methods^5^. By addressing these deficiencies and encouraging an improved degree of exploration within each run, we find that our algorithms are able to more frequently locate good solutions for many targets as compared to Rosetta, provided that the fragment libraries employed are of sufficient quality. An advantage of being able to more frequently access good structures is that one needs fewer runs of a prediction technique in order to get structures of a desired quality^47,48^. Improved distributions of predictive accuracy in decoy sets can also translate into improvements following typical model selection procedures, as clustering procedures are more likely to detect near-native states if these are sufficiently populated.

The improvements we see over Rosetta stem primarily from the combination of strategies of forced perturbation and local search that our methods employ. Together, these strategies encourage a relatively high degree of movement in conformational space within individual runs and also enable the search to visit shallow local optima in the energy landscape. The use of a simulated annealing framework further helps exploration, by allowing more energetic structural states to be transiently visited, particularly near the start of each low-resolution stage employing this framework. This encourages transitions between structurally distinct regions of the search space and allows each run of our protocols to make more informed decisions about promising solutions.

Comparing our two protocols, we find that with a few exceptions, the ILS protocol shows comparatively better performance than the bilevel protocol, both in terms of predictive accuracy and score values obtained. Local and global measures of sampling performance indicate that the ILS protocol is also capable of an improved degree of conformational exploration, since the bilevel protocol may be misguided by inaccurate secondary structure predictions (Supplementary information). Such an effect has been demonstrated in our recent work^49^, where predicted SS was used to inform the search process.

Using our sampling protocols, we were able to observe striking differences in our ability to obtain good predictions for certain targets, when alternative fragment sets were used. This result has some important implications. In works describing new fragment-based search algorithms, these methods are frequently benchmarked using fragment sets that exclude fragments drawn from detectably homologous templates^6,19,23,24^. The motivation for doing so is to recapitulate truly *de novo* prediction scenarios in which no homologous structures are detectable. It appears that at least some sampling problems generated using such procedures may simply be too hard for current methods to solve; there may exist very few assemblages of fragments that correspond to native-like topologies, or in more extreme cases, none at all. In such cases, it is very likely that improved sampling methods may show improvements in terms of score/energy values obtained (as a result of improved optimisation), but these would not associate with improvements in predictive accuracy. Such trends have been observed in a number of studies^21,23,24,50^, as well as for some of the targets studied here. We speculate that at least some of these studies may have defined search problems that were simply too difficult to be useful for comparing search methods.

For current methods, a more useful testbed for comparing search methods can be provided when a fragment set for a given target can be shown to support native-like states and when it can be shown that these states can actually be reached by successive fragment insertion operations. The first condition is quite easy to satisfy in benchmarking scenarios, for example by controlling the number of native-like fragments in each insertion window. However, the second condition is much more difficult to ensure. One could envisage the possibility that a sampling method will occasionally generate structures that are simply an assemblage of the native-derived fragments, however, in general this is only possible if these fragments are strung together without any attention to score/energy values. In other words, in a typical prediction trajectory, most transitional structural states sampled on the path to assembling the native structure must be energetically favourable and this is very difficult, if not impossible, to guarantee. Said differently, near-native states may be separated from non-native ones by an energy barrier that is too high and wide for any heuristic making use of only energy values to pass over. The *Perturbation* steps in our sampling methods do ignore score values and so can allow the search to escape from local minima. However, most moves in our protocols still take score values into account and our archiving strategy also uses score values to select promising decoys. It may be valuable to investigate whether even more disruptive perturbation operators may provide advantages in difficult prediction problems.

Some recent work has investigated the properties of fragment sets that enable high-accuracy structure prediction^6,51,52^, as well as improved methods for fragment library generation^6,53–55^. Nevertheless, the question of how to generate fragment sets that define informative problems for benchmarking search protocols deserves further investigation. Using well-designed procedures for fragment library generation, it may be possible to generate test problems of varying difficulty for given target proteins, which would be valuable for assessing the effectiveness of different sampling strategies under different conditions of difficulty. This would be valuable in developing and benchmarking new sampling protocols that can tackle progressively more difficult prediction problems.

## Electronic supplementary material

Supplementary information

## Data Availability

Our methods are available as source code patches to Rosetta and can be downloaded from https://github.com/shaunmk/Bilevel-ILS-Rosetta. Rosetta 3.4 source code is required, which is free to academic users. The fragment sets used in this work are available on Zenodo (https://zenodo.org/record/1254031), along with additional data.
