# Supplementary material for: Improved fragment-based protein structure prediction by redesign of search heuristics
Source: Sci Rep. 2018 Sep 12;8:13694. doi: 10.1038/s41598-018-31891-8 (PMC6135816; doi:10.1038/s41598-018-31891-8)
Supplement: Supplementary file 1 — Supplementary information [file 41598_2018_31891_MOESM1_ESM.pdf]

Supplementary information: Improved fragment-based protein structure prediction by redesign of search heuristics

Shaun M. Kandathil, Mario Garza-Fabre, Julia Handl and Simon C. Lovell

S1 Detailed description of key components of the bilevel and ILS protocols

S1.1 Perturbation and local search steps

Our methods utilise a strategy of perturbation followed by quick local optimisation. The large perturbation step is carried out by performing a single fragment insertion and accepting this change regardless of its effect on the energy or score of the structure. This forms the basis of the *Perturbation* operator in Algorithm 1 in the main text. Following this, the *LocalSearch* operator performs a series of moves. The *LocalSearch* step only accepts changes that lead to a decrease in energy (this is termed greedy optimisation). The *LocalSearch* process is terminated when a certain number of hill-climbing moves have been attempted without any of them being accepted. Currently, we set this limit to 50 consecutive move attempts.

Once this limit is reached, the *LocalSearch* is said to have reached a local minimum (*LMin*) in the energy landscape. Setting the termination criterion for the *LocalSearch* steps in this manner implies that we consider relatively shallow basins in the energy landscape to be local minima. This is in contrast to the approach taken, for example, in Rosetta Abinitio, where after 150 consecutive rejected moves, the value of the temperature parameter is increased with a view to accept more "risky" moves; this corresponds to searching for deeper energy minima. We opted to use a relatively shallow descent into the energy landscape following the analysis by Molloy et al. (2013), who demonstrated that aggressive minimisation of the low-resolution Rosetta score functions can be detrimental to search performance, as it tends to trap the search in very deep, narrow local optima.

Once a local minimum (*LMin*) has been reached, the next step is to compare this *LMin* structure with the *LMin* last encountered. This comparison comprises the *AcceptanceCriterion* step in Algorithm 1. If an *LMin* is rejected by the *AcceptanceCriterion* step, the structure is reset to the last accepted *LMin* structure, and a new *Perturbation* move is performed. The comparison between successive *LMin* structures in each *AcceptanceCriterion* step is done on the basis of energy, using the Metropolis criterion (Metropolis et al., 1953). By this criterion, a new structural state generated by a move or series of moves is accepted with a probability P which is given by

$$P = \begin{cases} 1, & \text{if } \Delta E \leq 0 \\ \exp(\frac{-\Delta E}{kT}), & \text{if } \Delta E > 0 \end{cases} \text{, where}$$

ΔE is the change in score between the two *LMin* states being compared,
k is the Boltzmann constant, and
T is the thermodynamic temperature.

In Rosetta, the product kT is specified as a single parameter whose values are scaled according to typical score values seen. We vary the value of this term using a simple scheme of simulated annealing (Kirkpatrick et al., 1983), whereby the Metropolis temperature parameter is gradually decreased from an initial high value. We used this simulated annealing procedure for the *AcceptanceCriterion* step with a view to encourage more disruptive moves in the early parts of each low-resolution stage employing our sampling framework. We used initial and final values of 10 and 2 kT units, respectively, for the temperature parameter. The initial value of 10 was chosen based on the analysis by Bowman and Pande (2009), who used a simulated tempering method for conformational sampling together with the low-resolution scoring functions in Rosetta. Their analysis indicated that Rosetta temperature settings of 10-20 kT units were sufficient to realise a high degree of exploration for small proteins. To prevent the search process from devolving into a purely random search, we used an initial value of 10 kT units for the Metropolis temperature. The final value of 2 kT units corresponds to the standard setting in Rosetta, which leads to relatively conservative exploration. The temperature associated with any given *AcceptanceCriterion* step is a function of the initial and final temperature settings, as well as the predetermined total number of score function evaluations in the current low-resolution stage. The value of the temperature parameter is reset at the beginning of each low-resolution stage employing bilevel optimisation. The temperature value T at a given score function evaluation number t is given by an exponential cooling schedule:

$$T_t = \alpha^t \cdot T_i \text{ , where}$$

$$\alpha = \left(\frac{T_i}{T_f}\right)^{1/n} \quad ,$$

n is the number of score function evaluations in the current stage ($n \geq t$),
T_i is the initial temperature value, and
T_f is the final temperature value.

Thus, the annealing proceeds by exponential decay until the end of a given stage, when $t = n$, and here the value of the temperature parameter T_t equals T_f.

One potential problem with methods based on temperature control (such as simulated annealing) is that different proteins may require tailored balances between exploration and exploitation, and some parameter tuning may be necessary to achieve optimal results in individual cases. For example, QUARK (Xu and Zhang, 2012) sets run length and temperature settings for its replica exchange procedure as a function of target length. Similar strategies have been employed in FRAGFOLD (Jones et al., 2005; Kosciołek and Jones, 2014). Parameter adaptation is an area that can be investigated in future work.

We will now discuss the details of how the *Perturbation* and *LocalSearch* steps are implemented for each of our protocols.

S1.1.1 Implementation for the bilevel protocol

In the bilevel protocol, the *Perturbation* operator comprises a single fragment insertion in a segment of the protein structure containing loop residues, while the *LocalSearch* operator repeatedly alters non-loop residues. The *LocalSearch* operator forms the "lower-level" optimiser, while the "upper-level" optimiser can be seen as all components in lines 2-10 of Algorithm 1 in the main text, leaving out the *LocalSearch* step. The lower-level optimiser operates within the constraints set up by the upper-level optimiser. The constraints here correspond to the values of structural parameters

(torsion angles) set by the *Perturbation* step in the loop residues. The *AcceptanceCriterion* step corresponds to the acceptance criterion of the upper-level optimiser. Thus, following the usual approach taken in bilevel optimisation, a solution can be accepted by the upper-level optimiser only when it is an optimum in the lower-level optimisation problem.

The bilevel protocol depends on prior knowledge of which residues are involved in loop regions. Given a native structure, secondary structure (SS) is typically assigned using a classification algorithm such as DSSP (Kabsch and Sander, 1983). However, for the bilevel protocol to be useful in blind prediction, we must rely on a SS prediction program to provide these assignments. In this work we make use of PSIPRED (Jones, 1999) to provide SS assignments. For proof of principle, using a single SS prediction allows us to rigidly define the boundaries of SS elements and loops, and allows us to identify cases where inaccurate SS prediction may be an important factor affecting predictive ability. We used PSIPRED to provide the bilevel protocol with SS assignments, since we used PSIPRED as the sole SS prediction that is used to inform the choice of fragments available in the fragment set in each insertion window (Kandathil et al., 2016). The fragment picking process can alternatively be configured to use a quota system employing three prediction methods (Gront et al., 2011). The use of a consensus prediction from independent SS prediction programs, and/or frameworks that take the probabilities output by these methods into account, are all possibilities for future development.

Table 1 summarises the rules we defined for whether and how to apply a proposed fragment insertion in the *Perturbation* step of the bilevel protocol, depending on the arrangement of loops and SS elements in the current insertion window. These rules were defined so as to direct the search towards altering an appreciable number of loop residues in any single *Perturbation* step, and alternative sets of rules consistent with the bilevel framework can be devised. Terminal loops are excluded from the set of loops that can be altered by the *Perturbation* steps. This is done in order to prevent a large number of moves being spent altering these regions; we identified this behaviour as one potential cause of poor sampling performance in our previous work (Kandathil et al., 2016). Currently, we implement the SS-dependent move framework as a "filtering" step, after an insertion window has been chosen at random along the chain. In other words, once a fragment insertion has been proposed in the *Perturbation* step for a given insertion window, we evaluate the PSIPRED prediction for this window to determine if the fragment should be applied, according to the rules in Table 1. The *LocalSearch* steps are only allowed to change non-loop residues in any insertion window. The use of these rules leads to resultant fragment insertions with lengths that are less than or equal to the length of the fragments being used. It has been suggested that the use of such moves can provide some advantages in fragment-based structure prediction (Handl et al., 2012).

S1.1.2 Implementation for ILS protocol

The iterated local search (ILS) protocol can be seen as a modified version of the bilevel protocol, in which both the *Perturbation* and *LocalSearch* steps are allowed to alter any part of the chain. In the ILS protocol, the changes made to the structure by the *Perturbation* steps are not enforced as a constraint. In other words, the *LocalSearch* operator is allowed to affect residues that were altered by the *Perturbation* step, thus moving away from the bilevel framework in which this is not allowed. All move sizes in the ILS protocol are equal to the fragment length (9 residues), and each run of the ILS protocol uses the same total number of scoring function evaluations as a run of the bilevel protocol. All other aspects, such as the simulated annealing framework and its associated parameters, are kept identical.

Table S1: Rules for applying a proposed fragment insertion in the *Perturbation* steps, depending on the predicted local three-state secondary structure (SS) of the target protein. In the first column, the extents of the proposed fragment insertion are shown above a depiction of local secondary structure. Coloured boxes represent secondary structure elements such as α helices and β strands. These are joined by line segments, representing loops. The remaining two columns describe each condition, and the rule for accepting or rejecting the proposed move.

Illustration	Description	Accept or reject?
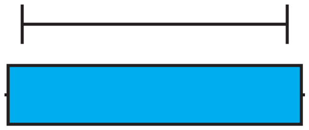	No loop residues in insert	Reject move
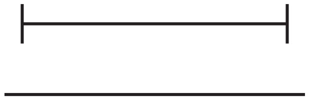	All residues in insert are loop	Accept move
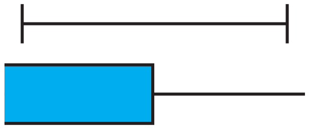	Single loop partially covered by insert	Accept changes to loop residues only
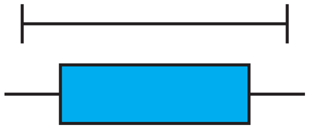	SS element spanned completely, but no loop spanned completely	Reject move
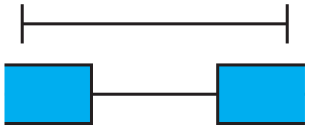	Single loop spanned completely, optionally with partially spanned SS elements at either end	Accept changes to loop residues only
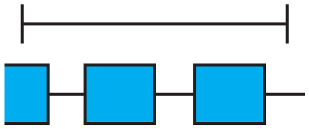	More than one loop spanned completely	Select one completely spanned loop at random and accept changes to that loop only

S1.2 Solution archiving

For both protocols, we keep and update a collection or archive of the best *LMin* structures encountered as the search proceeds (*UpdateArchive* in Algorithm 1 in the main text). Currently, we define the set of the "best" *LMin*s as those with the lowest Rosetta scores in the current stage. Once stored in this archive, solutions are not used to guide the search in any way, although this is another area that can be explored in future work. The purpose of storing a set of structures that meet some criteria is to try to retain multiple local minima that are potentially diverse in structure. This could be particularly useful in more difficult prediction scenarios, which are characterised by an energy landscape showing many distinct basins with nearly equal energy values. Although storing low-energy solutions does not guarantee that a structurally diverse set of solutions will be captured, we implemented a framework that allows us to employ different criteria to define the set of solutions that should be retained.

The desired size of the archive of structures is set in advance, and every *LMin* encountered during the search is considered for addition to the archive. We currently save the 10 best *LMin* structures encountered. The first set of consecutive *LMin*s encountered in stage 2 are added to the archive to create the initial solution set. Subsequently encountered *LMin* structures are added to the archive if their score is lower than that of the *LMin* with the highest score currently present in the archive. In this way, the archive is constantly updated to contain a set number of the lowest-scoring structures in any stage. Whenever the scoring function is changed during the search (e.g. when transitioning between low-resolution stages), all solutions in the archive are re-evaluated with the new scoring function, ensuring that any subsequent comparison between the archive and a new *LMin* is based on the same scoring function.

S2 Entropy data for all targets

Table S2: Median entropy and Median Absolute Deviations (MADs, $n = 8$ runs), comparing the ILS and bilevel protocols, as well as sets of short Rosetta runs. Data are shown for all targets, arranged in order of PDB identifier.

Target	ILS Protocol		Bilevel Protocol		Rosetta	
	Median	MAD	Median	MAD	Median	MAD
1a19A	0.3668	0.0201	0.3229	0.0167	0.1633	0.0451
1a32	0.3983	0.0219	0.4921	0.0078	0.3353	0.0249
1acf	0.2953	0.0200	0.2655	0.0146	0.1467	0.0205
1ail	0.4202	0.0276	0.3065	0.0231	0.6164	0.0040
1aiu	0.4367	0.0328	0.3587	0.0163	0.2641	0.0154
1b3aA	0.3618	0.0366	0.2985	0.0169	0.2162	0.0124
1bgf	0.4107	0.0434	0.2398	0.0217	0.2000	0.0236
1bk2	0.2706	0.0118	0.2443	0.0545	0.2316	0.0278
1bkrA	0.4813	0.0239	0.1249	0.0228	0.1922	0.0428
1bm8	0.3537	0.0213	0.2831	0.0260	0.2107	0.0148
1bq9A	0.3171	0.0132	0.3030	0.0287	0.2268	0.0155
1c8cA	0.4139	0.0097	0.3240	0.0098	0.3328	0.0342
1c9oA	0.3218	0.0269	0.2872	0.0263	0.3002	0.0351
1cc8A	0.2343	0.0073	0.1766	0.0328	0.1825	0.0110
1cei	0.4444	0.0386	0.1864	0.0183	0.2188	0.0071
1cg5B	0.3706	0.0155	0.1502	0.0261	0.2099	0.0335
1ctf	0.2455	0.0121	0.2451	0.0070	0.2205	0.0189
1dhn	0.2745	0.0365	0.2318	0.0379	0.1006	0.0193
1elwA	0.3101	0.0178	0.5130	0.0244	0.7422	0.0086
1enh	0.4617	0.0223	0.3437	0.0511	0.4376	0.0080
1ew4A	0.3954	0.0379	0.2900	0.0383	0.2152	0.0293
1eyvA	0.3780	0.0204	0.2016	0.0118	0.2277	0.0301
1fkb	0.3079	0.0311	0.2773	0.0252	0.1391	0.0215
1fna	0.3976	0.0296	0.3509	0.0204	0.0943	0.0159
1gvp	0.3215	0.0383	0.3171	0.0678	0.2410	0.0243
1hz6A	0.3218	0.0196	0.3987	0.0239	0.2714	0.0137
1ig5A	0.3151	0.0232	0.2377	0.0168	0.2893	0.0179
1iibA	0.3324	0.0394	0.3041	0.0197	0.1379	0.0120
1kpeA	0.3116	0.0315	0.2395	0.0476	0.1067	0.0176
1lis	0.2894	0.0212	0.2107	0.0528	0.2552	0.0186
1louA	0.3255	0.0500	0.2206	0.0208	0.1414	0.0159
1npsA	0.3665	0.0321	0.3096	0.0556	0.1786	0.0115
1opd	0.2438	0.0105	0.2356	0.0246	0.1787	0.0181
1pgx	0.4394	0.0166	0.3076	0.0305	0.3484	0.0258
1ptq	0.3229	0.0168	0.1973	0.0241	0.3272	0.0197
1r69	0.4853	0.0332	0.3824	0.0225	0.3912	0.0199
1rnbA	0.4773	0.0268	0.3471	0.0508	0.3969	0.0128
1scjB	0.4117	0.0208	0.3512	0.0387	0.2120	0.0100

continued...

Target	ILS Protocol		Bilevel Protocol		Rosetta	
	Median	MAD	Median	MAD	Median	MAD
1shfA	0.3067	0.0207	0.1839	0.0244	0.2696	0.0093
1ten	0.3761	0.0247	0.3079	0.0363	0.2131	0.0336
1tif	0.2917	0.0336	0.2367	0.0267	0.2892	0.0042
1tig	0.3419	0.0147	0.2247	0.0250	0.1721	0.0205
1tit	0.3833	0.0484	0.2944	0.0729	0.1190	0.0025
1tul	0.3429	0.0296	0.1780	0.0304	0.0783	0.0124
1ubi	0.5424	0.0175	0.4572	0.0121	0.3727	0.0408
1ughI	0.3794	0.0258	0.2588	0.0697	0.1927	0.0163
1urnA	0.4729	0.0197	0.3389	0.0300	0.1843	0.0158
1utg	0.2596	0.0393	0.1261	0.0231	0.2020	0.0154
1vcc	0.3921	0.0154	0.2752	0.0341	0.2447	0.0417
1vie	0.3273	0.0163	0.3687	0.0155	0.3004	0.0183
1who	0.4069	0.0601	0.2319	0.0154	0.1268	0.0127
1wit	0.3283	0.0223	0.2180	0.0318	0.1640	0.0037
256bA	0.4129	0.0153	0.3973	0.0298	0.1857	0.0102
2acy	0.2843	0.0350	0.1851	0.0221	0.1995	0.0127
2chf	0.3843	0.0151	0.3002	0.0348	0.1374	0.0256
2ci2I	0.2635	0.0100	0.2427	0.0154	0.2519	0.0164
2vik	0.3782	0.0178	0.1980	0.0157	0.1489	0.0167
4ubpA	0.0662	0.0065	0.0601	0.0051	0.1945	0.0157
5croA	0.3505	0.0049	0.4226	0.0152	0.3054	0.0154

S3 Results following all-atom refinement and clustering

Figure S1 shows distributions of Cα RMSD from the native for a total of 50,000 all-atom decoys generated per target and sampling protocol. The sampling protocols in this data made use of the newer fragment sets. These distributions are highly similar to those seen pre-refinement (red distributions in Figure S2), suggesting that there is no widespread deterioration in decoy quality following refinement. Table S3 shows the RMSD values of the top cluster centre identified by Calibur from each decoy set. This table also has details about the 59 test proteins, together with SS class and length.

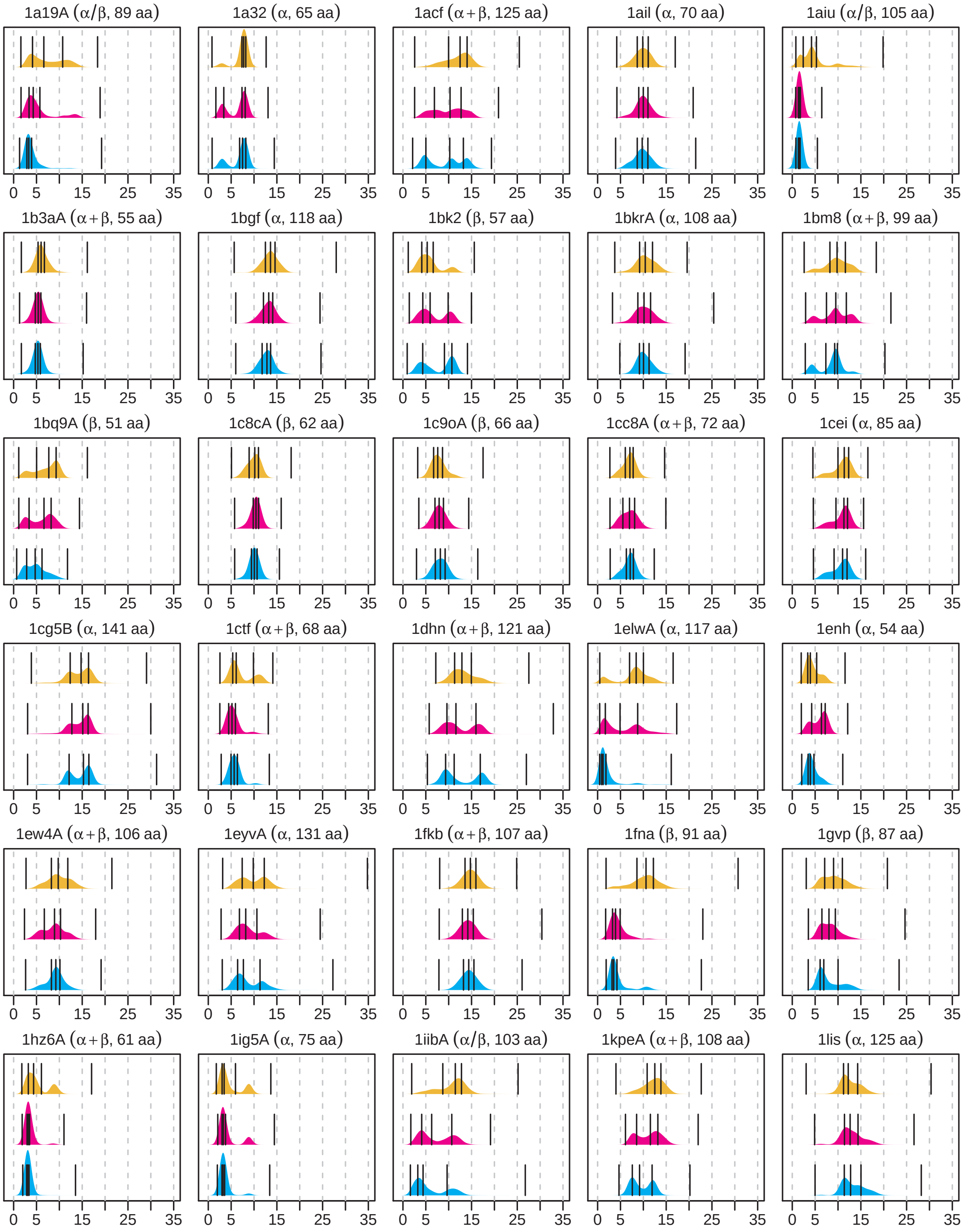

Figure S1: Kernel density plots of the distribution of Cα RMSD from the native (Ångströms, x-axis), for Rosetta (gold), the bilevel (magenta) and ILS protocol (blue), following all-atom refinement. PDB codes, SCOP secondary structure class and length in amino acid residues (aa) are given in the title of each plot. Targets are ordered by PDB identifier. Each distribution uses RMSD data from 50,000 decoys. Vertical lines represent the five-number summary (minimum, first quartile, median, third quartile and maximum) of each distribution (*figure continued on next page*).

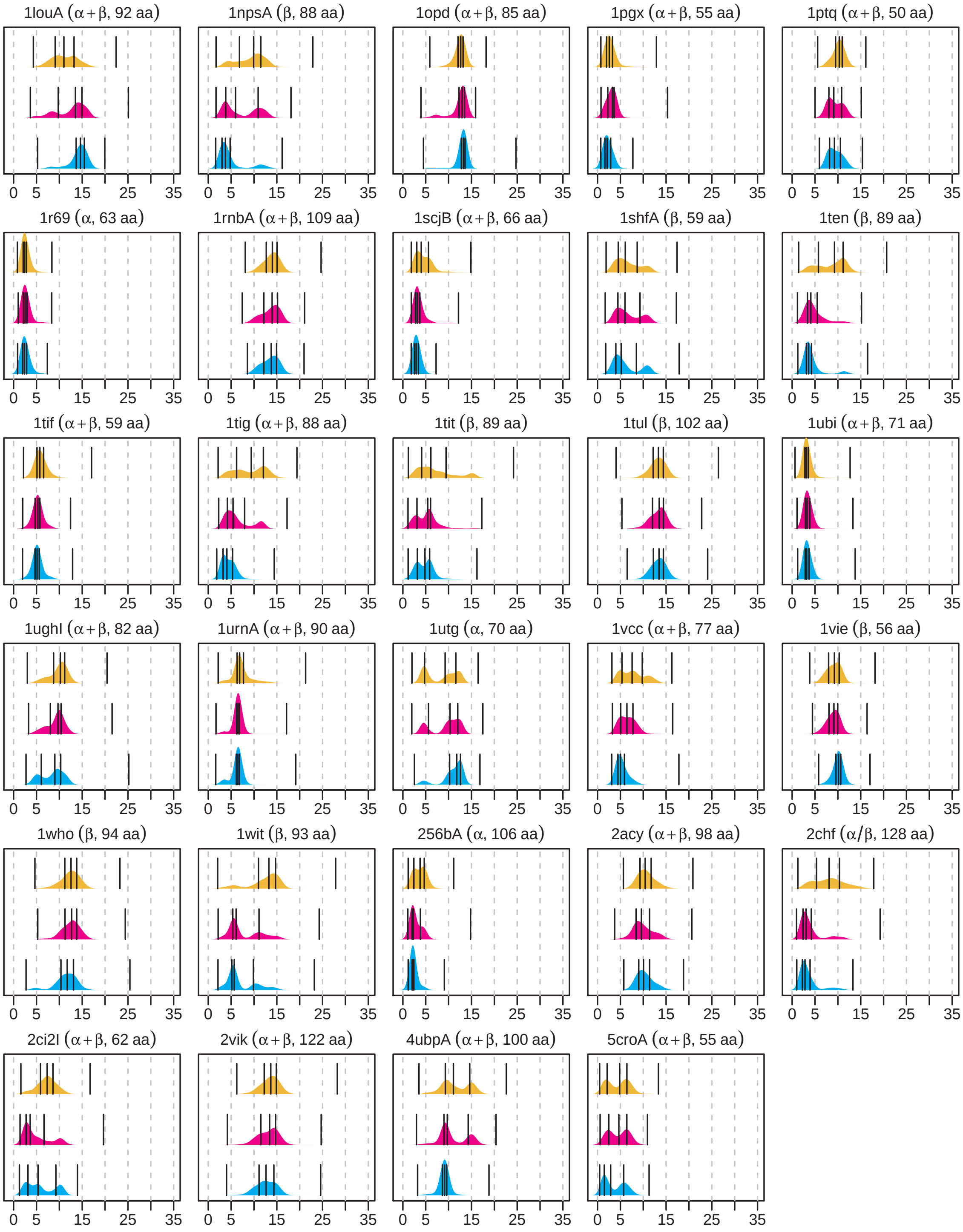

Figure S1 (*continued from previous page*).

Table S3: Cα RMSD from the native for all-atom predictions identified as the top cluster centre by Calibur, for each target and protocol, together with SCOP secondary structure class (SS) and length in residues. Proteins are sorted by PDB identifier. The lowest RMSD in each row is highlighted in bold face.

Target	SS	Length	Rosetta	Bilevel	ILS
1a19A	α/β	89	3.19	**2.71**	2.85
1a32	all-α	65	7.43	**2.89**	7.66
1acf	$\alpha+\beta$	125	8.52	4.66	**3.60**
1ail	all-α	70	7.96	8.58	**7.87**
1aiu	α/β	105	1.59	**1.09**	**1.09**
1b3aA	$\alpha+\beta$	55	5.17	5.02	**4.48**
1bgf	all-α	118	**11.64**	12.40	12.11
1bk2	all-β	57	3.58	3.34	**3.33**
1bkrA	all-α	108	7.95	**6.96**	8.40
1bm8	$\alpha+\beta$	99	6.84	**3.96**	9.62
1bq9A	all-β	51	3.65	2.83	**2.70**
1c8cA	all-β	62	**7.87**	10.00	9.91
1c9oA	all-β	66	**6.79**	7.34	7.25
1cc8A	$\alpha+\beta$	72	**6.79**	7.24	7.13
1cei	all-α	85	11.65	11.77	**11.63**
1cg5B	all-α	141	11.62	15.72	**11.42**
1ctf	$\alpha+\beta$	68	5.17	**4.30**	5.36
1dhn	$\alpha+\beta$	121	8.80	**8.46**	8.52
1elwA	all-α	117	8.07	1.19	**0.56**
1enh	all-α	54	**3.15**	7.19	3.45
1ew4A	$\alpha+\beta$	106	**8.01**	9.17	9.28
1eyvA	all-α	131	5.58	**4.92**	5.85
1fkb	$\alpha+\beta$	107	14.49	**13.83**	14.93
1fna	all-β	91	**2.10**	2.61	3.06
1gvp	all-β	87	5.95	8.90	**5.63**
1hz6A	$\alpha+\beta$	61	**2.72**	2.94	2.75
1ig5A	all-α	75	**2.54**	2.66	2.97
1iibA	α/β	103	4.31	3.01	**2.69**
1kpeA	$\alpha+\beta$	108	9.26	6.75	**6.50**
1lis	all-α	125	11.14	11.47	**10.95**

Target	SS	Length	Rosetta	Bilevel	ILS
1louA	$\alpha+\beta$	92	**11.28**	14.51	15.25
1npsA	all-β	88	6.14	3.09	**2.33**
1opd	$\alpha+\beta$	85	**12.05**	13.06	13.40
1pgx	$\alpha+\beta$	55	**1.50**	3.45	1.58
1ptq	$\alpha+\beta$	50	10.59	7.96	**6.98**
1r69	all-α	63	**1.88**	2.02	1.90
1rnbA	$\alpha+\beta$	109	**10.65**	16.37	11.14
1scjB	$\alpha+\beta$	66	2.88	2.60	**2.25**
1shfA	all-β	59	3.64	3.55	**3.28**
1ten	all-β	89	4.79	3.42	**3.08**
1tif	$\alpha+\beta$	59	**5.03**	5.31	5.60
1tig	$\alpha+\beta$	88	4.09	**2.87**	3.10
1tit	all-β	89	**2.43**	5.56	5.64
1tul	all-β	102	**13.82**	14.24	14.38
1ubi	$\alpha+\beta$	71	**2.63**	3.18	2.84
1ughI	$\alpha+\beta$	82	5.34	10.24	**5.33**
1urnA	$\alpha+\beta$	90	6.40	6.20	**6.13**
1utg	all-α	70	**4.10**	4.77	10.26
1vcc	$\alpha+\beta$	77	4.44	4.66	**4.15**
1vie	all-β	56	10.29	**9.36**	10.16
1who	all-β	94	11.66	13.12	**8.89**
1wit	all-β	93	13.86	**5.01**	5.31
256bA	all-α	106	1.96	**1.76**	1.92
2acy	$\alpha+\beta$	98	8.79	8.99	**8.03**
2chf	α/β	128	4.49	1.70	**1.66**
2ci2I	$\alpha+\beta$	62	4.94	**1.99**	2.35
2vik	$\alpha+\beta$	122	15.31	14.19	**8.74**
4ubpA	$\alpha+\beta$	100	15.01	9.12	**8.71**
5croA	$\alpha+\beta$	55	1.24	1.87	**0.93**

S4 Comparison of predictive accuracy achieved by three protocols using different fragment sets

Figure S2 shows the influence of fragment library quality on RMSD distributions, comparing Rosetta and our protocols, each using either the older or newer fragment sets for a given target. For the bilevel protocol, each run made use of the SS prediction generated with the respective fragment set. The red distributions of RMSD in this figure are generated using the same data in Figure 3 in the main text.

S5 The ILS protocol is more robust to inaccurate secondary structure predictions than the bilevel protocol

The key difference between the bilevel and ILS protocol is that the bilevel protocol makes use of predicted secondary structure (SS) information to inform how fragment insertions are carried out, whereas the ILS protocol does not use such information. Typically, both of our protocols show similar performance in terms of predictive accuracy distributions. However, from Figure S2, it can be seen that when the older fragment set for the target 1c8cA is used, the ILS protocol realises a much more favourable distribution of predictive accuracy than the bilevel protocol. The bilevel protocol does not achieve such an improvement, in contrast to the usually comparable results realised by both of our protocols.

The target 1c8cA has poorly-predicted SS assignments, in both the older and newer fragment sets (Figure S3, top row). Since the bilevel protocol relies on these SS assignments, we hypothesised that the poor SS prediction could be a reason for the reduced performance realised by the bilevel protocol for this target, with the older fragment set. We therefore ran another round of predictions using the “true” SS assignment for 1c8cA instead of the PSIPRED prediction. The true assignment was derived from the 8-state DSSP assignment for the native structure, which was converted to a 3-state assignment using the same procedure used to generate training data for PSIPRED, thus simulating a 100% accurate PSIPRED assignment. The results of this set of prediction runs are shown in the middle row of Figure S3. When the correct SS assignment is used, the bilevel protocol more closely matches the performance of the ILS protocol, in terms of both RMSD values, and score values. Thus, poor SS prediction can limit the predictive performance of the bilevel protocol. Because the ILS protocol does not make use of predicted SS information during the search, it is more robust to inaccuracies in SS prediction. However, since SS predictions are utilised during fragment picking, the ILS protocol may not perform well in a scenario where inaccurate SS prediction strongly biases a fragment set away from native-like structures. Inaccurate SS prediction does not automatically result in fragment sets incompatible with near-native structures, as is evident from the case of the target 1c8cA discussed here. In Rosetta, SS predictions are just one factor taken into account when choosing fragments. Local sequence and structure profiles tend to have a stronger influence on the choice of fragments in any window, and so fragment sets can be compatible with near-native structures even if the predicted SS is incorrect. It may be possible to improve robustness to poor SS prediction in the bilevel protocol by evaluating consensus between predictions from multiple SS prediction methods, and possibly utilising confidence scores reported by these methods.

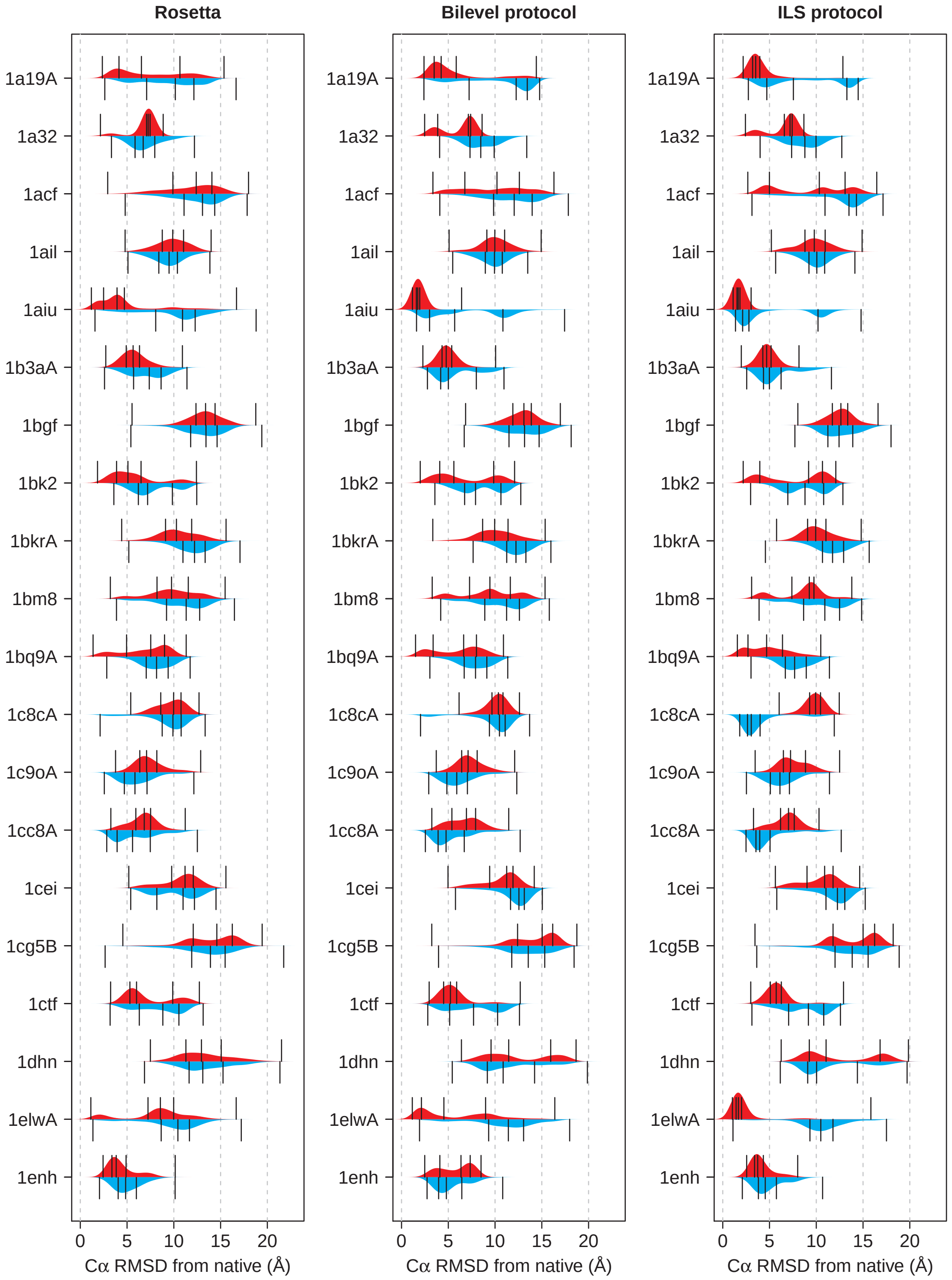

Figure S2: Kernel density plots of Cα RMSD from the native structure for all 59 targets, comparing decoy sets generated using the older and newer fragment libraries (blue and red distributions, respectively), for Rosetta, the bilevel protocol and the ILS protocol (left, centre and right panels, respectively). Each distribution comprises data from 1000 low-resolution decoys sampled using identical scoring function setups. Vertical lines represent the five-number summary of each distribution (*figure continued on next two pages*).

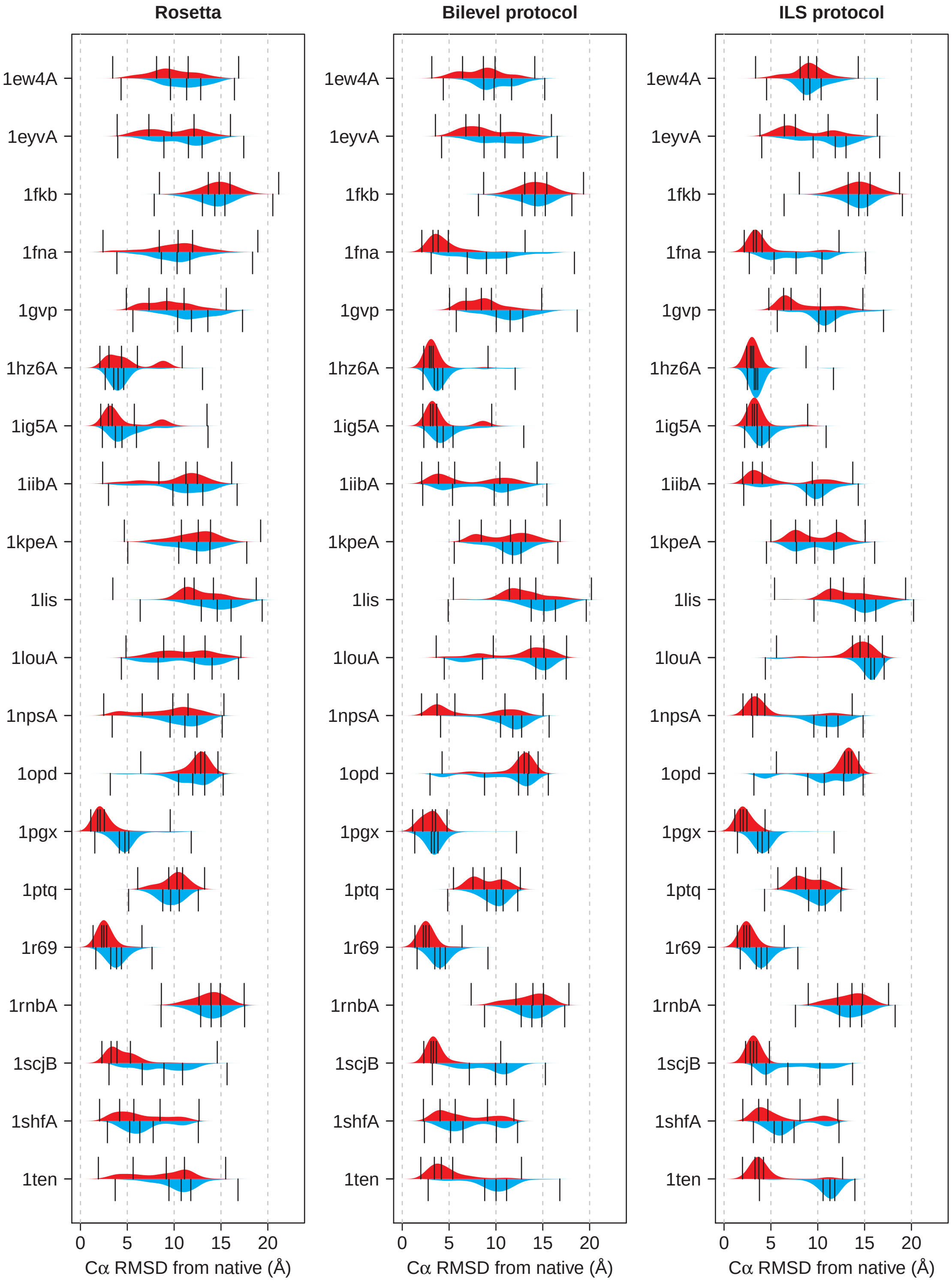

Figure S2 (*continued*)

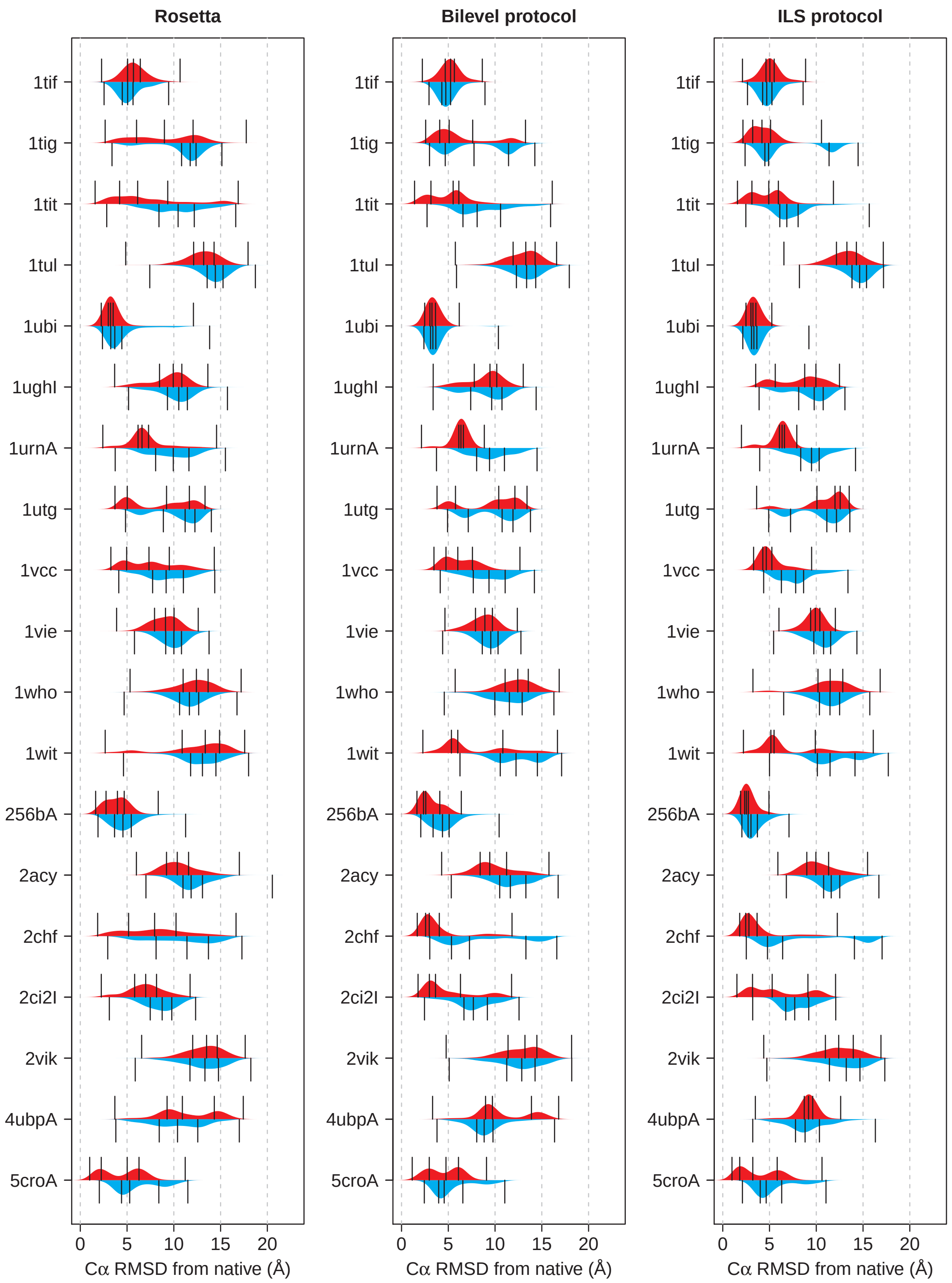

Figure S2 (*continued*)

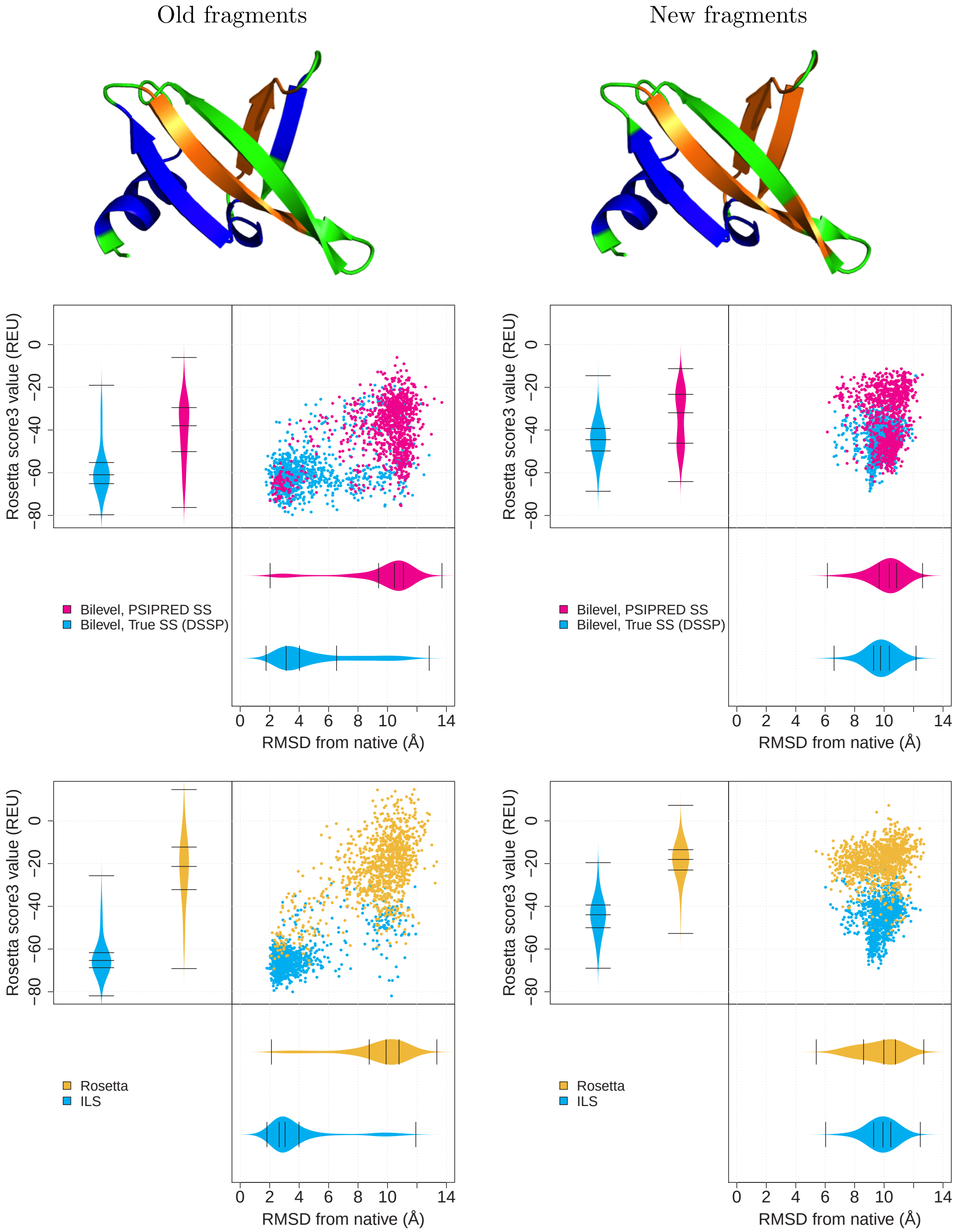

Figure S3: Effects of fragment library and SS prediction on predictive performance on target 1c8cA. The left and right columns compare data generated using older and newer fragment sets, respectively. **Top row:** Cartoon representation of the native structure, coloured according to PSIPRED SS assignments derived while generating the respective fragment set. Colours represent regions predicted as α helices (blue), β strands (orange), and loops (green). **Middle row:** Plots of Rosetta *score3* value in Rosetta Energy Units (REU) versus Cα RMSD from the native, comparing the performance of the bilevel protocol when using old and new fragments, and when given PSIPRED or DSSP SS assignments. Distributions of score and RMSD values are also shown as kernel density 'beanplots' with 5-number summaries marked. In the latter case, the bilevel protocol is able to much more reliably locate native-like decoys with the old fragment set, almost matching the ILS protocol on performance. The same improvement is not seen when the newer fragments are used, suggesting that the new fragment set is less enriched for native-like states. **Bottom row:** Score versus Cα RMSD plots for Rosetta and the ILS protocol; these protocols do not use SS information during prediction. Each dataset for each protocol comprises 1000 low-resolution decoys. NB Portions of the bottom two figures appear in a paper submitted to the 2017 Genetic and Evolutionary Computation Conference Companion (Handl et al., 2017).

Bibliography

Bowman, G. R. and Pande, V. S. (2009). Simulated tempering yields insight into the low-resolution Rosetta scoring functions. *Proteins: Structure, Function, and Bioinformatics*, 74(3):777–788.

Gront, D., Kulp, D. W., Vernon, R. M., Strauss, C. E. M., and Baker, D. (2011). Generalized fragment picking in Rosetta: Design, protocols and applications. *PLoS ONE*, 6(8):e23294.

Handl, J., Garza-Fabre, M., Kandathil, S. M., and Lovell, S. C. (2017). On heuristic bias in fragment-assembly methods for protein structure prediction. In *GECCO '17: Genetic and Evolutionary Computation Conference Companion*. ACM. In press. `doi:10.1145/3067695.3082545`.

Handl, J., Knowles, J., Vernon, R., Baker, D., and Lovell, S. C. (2012). The dual role of fragments in fragment-assembly methods for *de novo* protein structure prediction. *Proteins: Structure, Function, and Bioinformatics*, 80(2):490–504.

Jones, D. T. (1999). Protein secondary structure prediction based on position-specific scoring matrices. *Journal of Molecular Biology*, 292(2):195–202.

Jones, D. T., Bryson, K., Coleman, A., McGuffin, L. J., Sadowski, M. I., Sodhi, J. S., and Ward, J. J. (2005). Prediction of novel and analogous folds using fragment assembly and fold recognition. *Proteins: Structure, Function, and Bioinformatics*, 61(S7):143–151.

Kabsch, W. and Sander, C. (1983). Dictionary of protein secondary structure: Pattern recognition of hydrogen-bonded and geometrical features. *Biopolymers*, 22(12):2577–2637.

Kandathil, S. M., Handl, J., and Lovell, S. C. (2016). Toward a detailed understanding of search trajectories in fragment assembly approaches to protein structure prediction. *Proteins: Structure, Function, and Bioinformatics*, 84(4):411–426.

Kirkpatrick, S., Gelatt, C. D., and Vecchi, M. P. (1983). Optimization by simulated annealing. *Science*, 220(4598):671–680.

Kosciołek, T. and Jones, D. T. (2014). *De novo* structure prediction of globular proteins aided by sequence variation-derived contacts. *PLoS ONE*, 9(3):e92197.

Metropolis, N., Rosenbluth, A. W., Rosenbluth, M. N., Teller, A. H., and Teller, E. (1953). Equation of state calculations by fast computing machines. *The Journal of Chemical Physics*, 21(6):1087–1092.

Molloy, K., Saleh, S., and Shehu, A. (2013). Probabilistic search and energy guidance for biased decoy sampling in *ab initio* protein structure prediction. *IEEE/ACM Transactions on Computational Biology and Bioinformatics*, 10(5):1162–1175.

Xu, D. and Zhang, Y. (2012). *Ab initio* protein structure assembly using continuous structure fragments and optimized knowledge-based force field. *Proteins: Structure, Function, and Bioinformatics*, 80(7):1715–1735.
